# Novel Web-Based Drop-In Mindfulness Sessions (Pause-4-Providers) to Enhance Well-Being Among Health Care Workers During the COVID-19 Pandemic: Descriptive and Qualitative Study

**DOI:** 10.2196/43875

**Published:** 2024-03-14

**Authors:** Mary Elliott, Camille Khallouf, Jennifer Hirsch, Diane de Camps Meschino, Orit Zamir, Paula Ravitz

**Affiliations:** 1 Department of Supportive Care Princess Margaret Cancer Centre University Health Network Toronto, ON Canada; 2 Department of Psychiatry Faculty of Medicine University of Toronto Toronto, ON Canada; 3 Department of Psychiatry Sinai Health System Toronto, ON Canada; 4 Women's College Hospital Toronto, ON Canada; 5 Toronto Academic Pain Medicine Institute Women's College Hospital Toronto, ON Canada; 6 Toronto Rehabilitation Institute University Health Network Toronto, ON Canada; 7 Lunenfeld-Tanenbaum Research Institute Sinai Health System Toronto, ON Canada

**Keywords:** COVID-19, pandemic, health care worker, resilience, mental health, burnout, well-being, mindfulness meditation, web-based group, drop-in, mindfulness, health care staff, meditation, worker, job, occupational health

## Abstract

**Background:**

The COVID-19 pandemic exerted extraordinary pressure on health care workers (HCWs), imperiling their well-being and mental health. In response to the urgent demand to provide barrier-free support for the health care workforce, Pause-4-Providers implemented 30-minute live web-based drop-in mindfulness sessions for HCWs.

**Objective:**

This study aims to evaluate the use, feasibility, satisfaction, and acceptability of a novel mindfulness program aimed at enhancing the well-being of HCWs during the COVID-19 pandemic.

**Methods:**

Accrual for the study continued throughout the first 3 pandemic waves, and attendees of ≥1 session were invited to participate. The evaluation framework included descriptive characteristics, including participant demographics, resilience at work, and single-item burnout scores; feedback questionnaires on reasons attended, benefits, and satisfaction; qualitative interviews to further understand participant experience, satisfaction, benefits, enablers, and barriers; and the number of participants in each session summarized according to the pandemic wave.

**Results:**

We collected descriptive statistics from 50 consenting HCWs. Approximately half of the participants (24/50, 48%) attended >1 session. The study participants were predominantly female individuals (40/50, 80%) and comprised physicians (17/50, 34%), nurses (9/50, 18%), and other HCWs (24/50, 48%), who were largely from Ontario (41/50, 82%). Of 50 attendees, 26 (52%) endorsed feeling burned out. The highest attendance was in May 2020 and January 2021, corresponding to the first and second pandemic waves. The participants endorsed high levels of satisfaction (43/47, 92%). The most cited reasons for attending the program were to relax (38/48, 79%), manage stress or anxiety (36/48, 75%), wish for loving kindness or self-compassion (30/48, 64%), learn mindfulness (30/48, 64%), and seek help with emotional reactivity (25/48, 53%). Qualitative interviews with 15 out of 50 (30%) participants identified positive personal and professional impacts. Personal impacts revealed that participation helped HCWs to relax, manage stress, care for themselves, sleep better, reduce isolation, and feel recognized. Professional impacts included having a toolbox of mindfulness techniques, using mindfulness moments, and being calmer at work. Some participants noted that they shared techniques with their colleagues. The reported barriers included participants’ needing time to prioritize themselves, fatigue, forgetting to apply skills on the job, and finding a private place to participate.

**Conclusions:**

The Pause-4-Providers participants reported that the web-based groups were accessible; appreciated the format, content, and faculty; and had high levels of satisfaction with the program. Both novel format (eg, drop-in, live, web-based, anonymous, brief, and shared activity with other HCWs) and content (eg, themed mindfulness practices including micropractices, with workplace applications) were enablers to participation. This study of HCW support sessions was limited by the low number of consenting participants and the rolling enrollment project design; however, the findings suggest that a drop-in web-based mindfulness program has the potential to support the well-being of HCWs.

## Introduction

### Background

The COVID-19 pandemic has jeopardized the mental health and well-being of health care workers (HCWs). Rapidly shifting public health guidelines have been implemented in response to the emergent scientific findings. The uncertain landscape of this pandemic has produced a sense of uncertainty, fear, and psychological distress in HCWs [1]. This was compounded by the realities HCWs faced, including inequitable access to rationed resources such as personal protective equipment and needed acute care beds [2]. Changes in procedural guidelines and service priorities, ramping down of nonessential services, and shifting standards of care have contributed to moral distress [3-5]. Risk factors for HCWs’ burnout included heavy workload, shifting roles, need for additional training, redeployment, and hybrid models of web-based care with feeling disconnected from their usual support and community of practice [6,7]. Greater COVID-19 exposure risks forced many HCWs into isolation owing to fears of infecting family members [1,8,9]. Early in the pandemic, significant levels of fatigue, insomnia, anxiety, depression, and psychological distress among HCWs were reported [8,10-15]. Informed by the literature on previous pandemics, it was anticipated that as the pandemic endured, moral distress, posttraumatic stress, and burnout would become problematic [16]. All combined, the COVID-19 pandemic amplified the challenges faced by HCWs within an already-strained workforce and was thus critically important to address [1,3,9,17-22].

Before the pandemic, evidence-based mindfulness interventions for HCWs were found to enhance stress management, awareness, emotion regulation, connection, self-compassion, empathy, and resilience [23-27]. Research demonstrates that mindfulness training for HCWs can improve well-being by promoting self-care and cultivating self-compassion [28]. The term “mindfulness” is defined as the “awareness that arises through paying attention, on purpose, in the present moment, and nonjudgmentally” and implies the cultivating of compassion [29]. Most interventions for HCWs evolved from manualized formats of mindfulness-based stress reduction, mindfulness-based cognitive therapy, and mindful self-compassion, which require a large time commitment [29-31].

“Pause-4-Providers” was created during the first wave of the COVID-19 pandemic to support HCWs and be accessible using a web-based drop-in platform. This extended the program’s reach to underserved areas and those in quarantine, with adherence to physical distancing requirements [17,21,32-34]. To establish a safe space for weary HCWs, no registration was required, and anonymity was allowed as we did not require participants to turn on their cameras or introduce or disclose information about themselves [35]. Unique to this program was the flexible curriculum, making it adaptable to the varied and changing needs of HCWs with time [36]. We implemented Pause-4-Providers drop-in sessions at the beginning of the COVID-19 pandemic to provide momentary refuge and respite from the intensity associated with working in health care during the pandemic.

### Objective

The objective of this study was to evaluate the implementation of Pause-4-Providers, a 30-minute evening web-based drop-in mindfulness program for HCWs during the COVID-19 pandemic. We designed a descriptive and qualitative study to examine the feasibility, satisfaction, and experiences of the participants. The research questions for the descriptive study were as follows:

1. What is the feasibility and acceptability of the web-based drop-in mindfulness program?
2. What is the level of burnout and resilience among the consenting participants?

Research questions for the qualitative study included the following: What were the experiences, enablers, and barriers to participating in the Pause-4-Providers program?

## Methods

### Program Development and Format

Within the first weeks of the COVID-19 pandemic in Canada, our team of university-affiliated psychiatrists (ME, JH, DdCM, and OZ) with expertise in mindfulness met and collaboratively decided to offer free, 30-minute live web-based drop-in mindfulness sessions for HCWs with the objective of supporting their well-being. We defined HCWs as anyone working in a health care or social care setting, including those providing direct patient-facing services and those performing indirect roles. Along with health care providers and trainees, the sessions were open to those providing administrative facilities, those working in support services, and research staff. Our sessions used a secure, privacy-compliant Zoom (Zoom Video Communication, Inc) platform multiple times per week, starting on March 24, 2020. We e-blasted Pause-4-Providers program announcements to >400 health care institutions, hospitals, long-term care homes, shelters, university departments, and associations (eg, Ontario Medical Association, Royal College of Physicians and Surgeons of Canada, Canadian Mental Health Association, and Registered Practical Nurses Association of Ontario) for distribution of session links to their HCWs.

**Figure 1:**
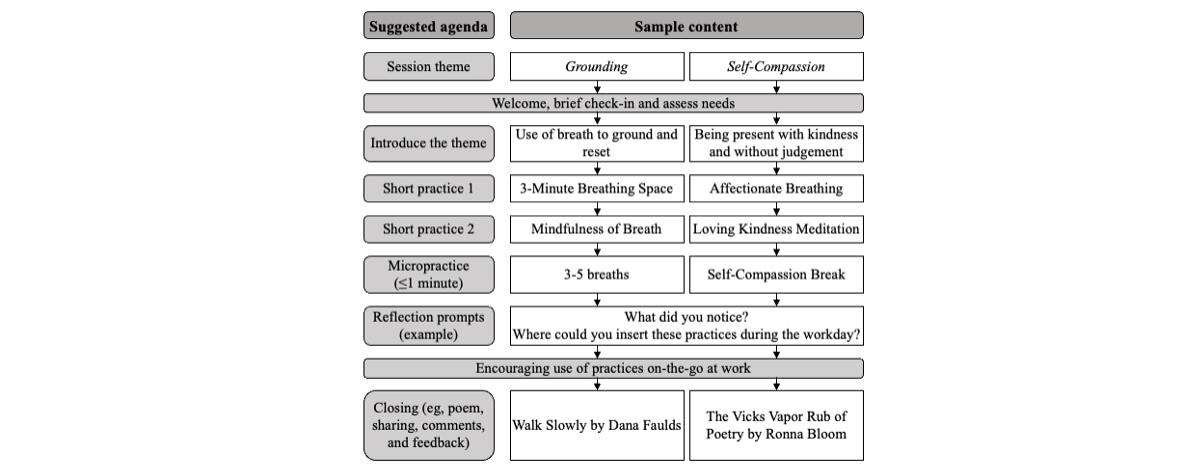
Sample agenda for Pause-4-Providers sessions.

Our group of faculty facilitators had extensive mindfulness training and clinical experience in leading manualized mindfulness group interventions in health care settings for HCWs and patients in an oncology health setting (ME) [21,35,37], patients with pain disorders (OZ), women with mental health concerns (DdCM and JH), and hospital-based health leaders and policy makers (DdCM). As mental health professionals working in hospital settings, we were also attuned to the psychological impacts the unfolding public health crisis had on HCWs. Notably, because we used the term “facilitators” when referring to the faculty who led the web-based drop-in sessions, we chose to use the term “enablers” when referring to factors in the qualitative analysis that facilitated or promoted participation. The themed agenda for each drop-in session was based on mindfulness, compassion, or resiliency skills to fit the emerging needs of HCWs, distressing local and global events and the time of the year or holidays. An evolving curriculum allowed for flexibility and responsiveness to the changing needs of HCWs [36]. We canvassed attendees using a situational needs assessment with routine quick check-ins at the start of each session to tailor the agenda accordingly. Given the time pressures and competing demands placed on HCWs, the sessions were intentionally designed to be of short duration. Each 30-minute session followed a semistructured agenda as presented in Figure 1 and Multimedia Appendix 1 [27,28,38,39]. The session themes evolved to reflect the immediate needs of HCWs, with a curricular structure of the sessions that was maintained. This structure included 2 short practices, including a micropractice and a closing. Maintaining a consistent format, the sessions introduced different practices and themes that were aligned with the objectives and principles of mindfulness and self-compassion. In small doses, with time, during the prolonged pandemic, faculty facilitators introduced a wide variety of mindfulness practices based on HCW attendees’ needs and levels of emotions (eg, hyperarousal vs emotional numbing). Drawing from positive psychology and resilience factors [40-45], the faculty facilitators used mindfulness techniques to promote positive emotions, compassion, and coping in the midst of challenges. Sessions included at least 1 guided micropractice (ie, a practice that was distilled into ≤1 minute meant for integration by participants throughout their workday) to emphasize how participants could apply aspects of the practices for self-care moments in the workplace [35,44,46,47]. Many sessions concluded with poems, followed by invited reflections and feedback. To support the program, we also created a website for Pause-4-Providers, where HCWs could find the schedule and session links, and access 4 prerecorded mindfulness practices for use at any time and participate in the research study.

### Ethical Considerations

This study was approved by the Sinai Health Research Ethics Board (20-0158-E). On the project website, participants were given a complete description of the study, and informed consent was obtained. The research assistant emailed the respondents who consented to be contacted for participation in a semistructured interview and obtained consent before the start of the interview. Participation in the study was voluntary and anonymous. The interviews were transcribed and deidentified to protect the identity of the study participants. The participants did not receive any compensation.

### Research Methods

This study aimed to evaluate the feasibility and acceptability of a web-based drop-in mindfulness program. We assessed feasibility quantitatively with attendance and qualitatively by exploring participants’ experiences of barriers and facilitators or enablers of attending such a program. We assessed acceptability using a brief satisfaction questionnaire and semistructured interviews with a subset of participants to explore their experiences of the program sessions. The burnout and resilience measures characterized the sample to learn about the potential suitability or appropriateness of the Pause-4-Providers program [48].

The study was open to all HCWs who attended ≥1 Pause-4-Providers session. Faculty facilitators informed HCW attendees in the sessions that they could visit the project website if they wished to participate or learn more about the research study. On the website, a banner informed HCWs of the research study with a link to a consent form and the confidential questionnaire package. Study participants could choose to participate in the descriptive portion of the study or both the descriptive and qualitative studies. The descriptive measures were collected once at study enrollment and included a questionnaire package consisting of demographics (eg, profession, age, sex, province or country of origin, and primary care setting), burnout and resilience measures, and a self-report feedback questionnaire focused on the participants’ reasons for attending, satisfaction, and utility of the sessions. At the end of the questionnaire, respondents could consent to be contacted for participation in a semistructured interview about their experiences of the program. There were no exclusion criteria for this study.

### Descriptive Measures

The *Single-Item Burnout Measure* [49] instructed respondents to define burnout for themselves with the following question: “Overall, based on your definition of burnout, how would you rate your level of burnout?” Participants scored their responses on a 5-category ordinal scale. The self-defined burnout measure has been useful as an abbreviated burnout assessment tool in medical professionals [50,51]. The self-defined burnout item correlates with the emotional exhaustion subscale of the Maslach Burnout Inventory (*r*=0.64; *P*<.001), defining burnout as a score of ≥3, with a correlation of 0.79, sensitivity of 83.2%, specificity of 87.4%, and an area under the curve of 0.93 (SE 0.004) [49].

The *Resilience at Work Scale* consists of 25 items measuring 7 aspects of workplace resilience, focusing on everyday behaviors. Participants rated their responses on a Likert scale, ranging from 0 (strongly disagree) to 6 (strongly agree) [52]. The scale is a validated tool for measuring and developing resilience among HCWs, with 67% of variance and an acceptable confirmatory factor analysis model fit (goodness of fit 0.97, Tucker-Lewis Index 0.98).

The Participant Self-Report Feedback Questionnaire was developed by us and asked about the number of sessions attended, reasons and motivation to attend (eg, to relax, improve sleep, be in a community, and learn mindfulness), benefits of attending (eg, increased awareness of emotional states, enhanced coping skills, and improved capacity to work with your team on a day-to-day basis), and overall satisfaction with the web-based mindfulness sessions. There were several open-text questions where respondents could describe how Pause-4-Providers influenced their coping (eg, positive or negative), describe the impact the pandemic had on their role and job, and provide feedback on the Pause-4-Providers program as presented in Multimedia Appendix 2.

### Qualitative Methodology

#### Overview

To explore the participants’ experiences and views of the Pause-4-Providers program, we used a qualitative research design, which does not test or generate theory but instead gathers explicit insight into clinical or behavioral interventions [53,54]. Qualitative methods are able to gather detailed insights into what does and does not work well to reveal the impacts, enablers, and barriers of program participation [55]. This is widely used in health service research to evaluate the determinants and impacts of interventions. The study complied with the standards for reporting qualitative research in accordance with COREQ (Consolidated Criteria for Reporting Qualitative Research) [54]. All participants consented before the interviews. There was no prior relationship between the qualitative researcher (Anna R Gagliardi) and the participants.

#### Sampling and Recruitment

At the end of the web-based questionnaire, respondents were asked if they were willing to be contacted for subsequent participation in an interview. Using a convenience sampling technique, all interested participants were emailed and invited to be interviewed by the research assistant (CK). The study and recruitment continued until June 2021, coinciding with the first 3 waves of the COVID-19 pandemic as defined by Public Health Ontario and presented in Multimedia Appendix 3. We sampled across 3 time frames corresponding to waves 1, 2, and 3 of the COVID-19 pandemic in Canada, with equal numbers of participants in each wave. We sampled concurrently with data collection and qualitative analysis and ceased when thematic saturation was achieved.

#### Data Collection

The audio-recorded (17-38 min), telephone-based, transcribed interviews conducted by the research assistant (CK) included questions about expectations, impacts, enablers, barriers, and recommendations to improve the program (Multimedia Appendix 4). Participants were asked about their motivation to attend the program, their experience of the program sessions, associated benefits or challenges, and feedback or suggestions for the future. Interview transcripts were independently analyzed by Anna R Gagliardi (a PhD-trained consultant with expertise in qualitative research), following which the findings were reviewed by all members of the research team.

### Data Analysis

#### Descriptive Analysis

The total weekly attendees at drop-in sessions were tallied from April 20, 2020, to June 21, 2021, as shown in Multimedia Appendix 3; a single participant would count multiple times in these tallies if multiple sessions were attended. These totals were plotted against time, with the ends of the first 3 pandemic waves on August 14, 2020; April 1, 2021; and July 6, 2021, respectively. The remaining analyses pertained only to survey respondents. Counts and percentages were used to summarize their demographic characteristics. Responses to questions on burnout, motivation, and experience were summarized, and the motivation and experience scales were collapsed into 3 levels (ie, very much [significantly or definitely], somewhat, and not at all or a bit). The 7 Resilience at Work subscale scores and the total score were each calculated according to the scoring guide, a 2-tailed *t* test was used to compare the mean overall score to that from a study on the psychological impact of COVID-19 on >500 hospital workers by Maunder et al [56] (email, August 13, 2021), and a 95% CI for the mean difference in overall scores was calculated. The relationship between the number of sessions attended (1 vs ≥2) and respondents’ feedback on the benefits of attending was assessed by cross-tabulating the 2 variables and calculating the *P* value using a Fisher exact test. Participants with a missing value for a variable were omitted from the analyses of that variable.

#### Qualitative Analysis

We used content analysis to identify themes for inductive coding in the qualitative interviews. This was done through constant comparison to develop a preliminary codebook of themes and exemplar quotes (first-level coding) [53]. We shared themes for discussion with all members of the research team to verify them. For subsequent interview transcripts, the qualitative expert researcher (Anna R Gagliardi) performed coding to expand or merge themes (second-level coding), tabulated participant characteristics and data (eg, themes and quotes), and examined similarities or differences by participant sampling characteristics. The research team reviewed and discussed the findings and further grouped them into categories related to motivation and facilitators, which we refer to as enablers, barriers, and impacts associated with participation. The qualitative findings were triangulated using descriptive findings.

## Results

### Descriptive Results

**Figure 2:**
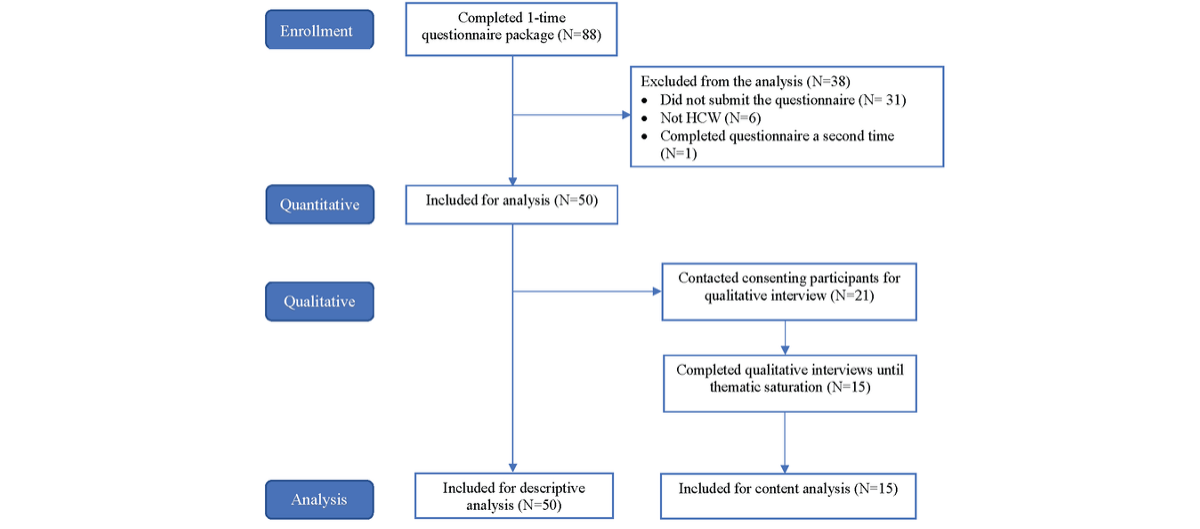
Participant CONSORT (Consolidated Standards of Reporting Trials) diagram. HCW: health care worker.

Between April 20, 2020, and June 27, 2021, there were 1333 attendance entries at the drop-in sessions. Consent was obtained from 88 respondents, most of whom (66/88, 75%) were enrolled during the second wave of the pandemic. Of 88 respondents, we excluded 38 (43%) from the analysis because they did not submit the questionnaire, were not HCWs, or completed the questionnaire a second time to arrive at a total sample of 50 (57%) participants, as presented in Figure 2. At the time of survey completion, 50% (25/50) had attended 1 session, 24% (12/50) 2 to 5 sessions, 8% (4/50) 6 to 10 sessions, and 16% (8/50) ≥11 sessions. If the participants indicated at the start of the questionnaire that they were completing it a second time, they were automatically taken to the end of the survey and only their first set of responses was included in the analysis.

Of 88 participants, the demographics were similar between the 50 (57%) included and 38 (43%) excluded participants. There were 5 sections in the survey on motivation, experience, satisfaction, burnout, and resilience. Overall, 90% (45/50) of the participants filled out all 5 sections, 10% (5/50) did not report on their prior experiences with mindfulness, and 6% (3/50) did not report on satisfaction. Of the 50 participants who submitted the survey, not all items or sections of the survey were completed. We included participants in the analysis if they provided data on ≥1 section of the questions we examined. Of the 50 HCWs, 24 (48%) had participated in >1 session; 17 (34%) were physicians, 9 (18%) were nurses, and the remaining 24 (48%) were other HCWs (eg, administrative or education assistant, occupational therapist, pharmacist, physiotherapist, psychotherapist, social worker, and others). Most respondents (40/50, 80%) were female individuals, 43% (21/50) were aged 20 to 39 years, 45% (22/50) 40 to 59 years, and 12% (6/50) >60 years, with participation primarily from the province of Ontario (41/50, 82%) and other Canadian provinces (eg, Alberta, British Columbia, New Brunswick, Quebec, and Saskatchewan).

Half of the HCWs (26/50, 52%) endorsed feeling burned out (score ≥3). Regarding Resilience at Work scores, participants scored highly (mean score >70) on the subscales of "finding your calling" (mean score 78, SD 17), "building networks" (mean score 78, SD 17), "living authentically" (mean score 76, SD 12), and "interacting cooperatively" (mean score 75, SD 15). Participants also scored moderately (mean score 50-69) on the workplace resilience subscale items of “staying healthy” (mean score 73, SD 22) and “managing stress” (mean score 65, SD 19) and reported difficulty with the subscale item “maintaining perspective” (mean score 48, SD 17). The overall mean resilience score was 69.4 (SD 12.6) [56].

In the self-report feedback questionnaire, participants’ most frequently reported motivations to attend the program included relaxing, managing stress or anxiety, wishing for loving kindness or self-compassion, and learning mindfulness, as presented in Table 1.

The most frequently endorsed benefits of attending included increased self-awareness of emotional states and reduced stress, as presented in Table 2. Participants who attended ≥2 sessions reported more positive benefits with coping skills (Fisher exact test *P*<.001) and managing stress (Fisher exact test *P*=.008) than participants who only attended 1 session. Of the 47 participants who reported their overall program satisfaction, 92% (43/47) rated it with a score of ≥4 on a 5-point Likert scale, with a mean score of 4.6 (SD 0.7).

**Table 1:** Self-reported motivation for attending the Pause-4-Providers sessions (N=48).

Motivation for attending the program	Very much or definitely motivated, n (%)	Moderately motivated, n (%)	Not at all or a bit motivated, n (%)
To relax	38 (79)	6 (13)	4 (8)
To manage stress or anxiety	36 (75)	9 (19)	3 (6)
Wish for loving kindness or self-compassion	30 (64)	9 (19)	8 (17)
To learn mindfulness	30 (64)	11 (23)	6 (13)
To seek help with emotional reactivity	25 (53)	9 (19)	13 (28)
To improve sleep	22 (47)	10 (21)	15 (32)
To gain energy	21 (45)	7 (15)	19 (40)
To learn tips to cope with work	20 (42)	12 (25)	16 (33)
To cope at home	19 (40)	12 (26)	16 (34)
To be in a community	14 (30)	12 (26)	21 (45)
To feel less isolated	10 (21)	9 (19)	28 (60)

**Table 2:** Self-reported benefits of attending the Pause-4-Providers sessions (N=45).

Benefits of attending the program	Very much or significantly benefited, n (%)	Somewhat benefited, n (%)	Not at all or a bit benefited, n (%)
Increased self-awareness of emotional states	25 (56)	12 (27)	8 (18)
Reduced stress level	24 (53)	12 (27)	9 (20)
Enhanced ability to be responsive rather than reactive to challenges as they arise	15 (33)	19 (42)	11 (24)
Enhanced coping skills	14 (31)	17 (38)	14 (31)
Improved capacity to work with teams on a day-to-day basis	11 (24)	15 (33)	19 (42)

### Qualitative Results

#### Participants

A total of 21 participants agreed to be contacted for participation in an interview and received a standardized invitation letter via email. Qualitative interviews were conducted with 15 consenting participants who answered the email and agreed to be interviewed, including 5 physicians, 2 administrative assistants, 2 physiotherapists, 2 psychotherapists, and 1 each of nurse practitioner, social worker, executive manager, and a speech pathologist. By age, this included 9 (60%) persons aged between 40 and 59 years and 6 (40%) 20 and 39 years. A considerable number of qualitative study respondents were women (13/15, 87%) than men, reflecting the fact that, out of 15 participants, 40 (80%) women and 7 (14%) men completed the descriptive questionnaire. Participants were evenly divided in terms of when they were interviewed during the COVID-19 pandemic: 5 (33%) in the first wave, 5 (33%) in the second wave, and 5 (33%) in the third wave. Interview participants completed a mean of 7 sessions (median 10, range 1 to 11) at the time of completing the questionnaire. Most participants had some prior exposure to mindfulness (11/15, 73%).

#### Themes

##### Overview

**Figure 3:**
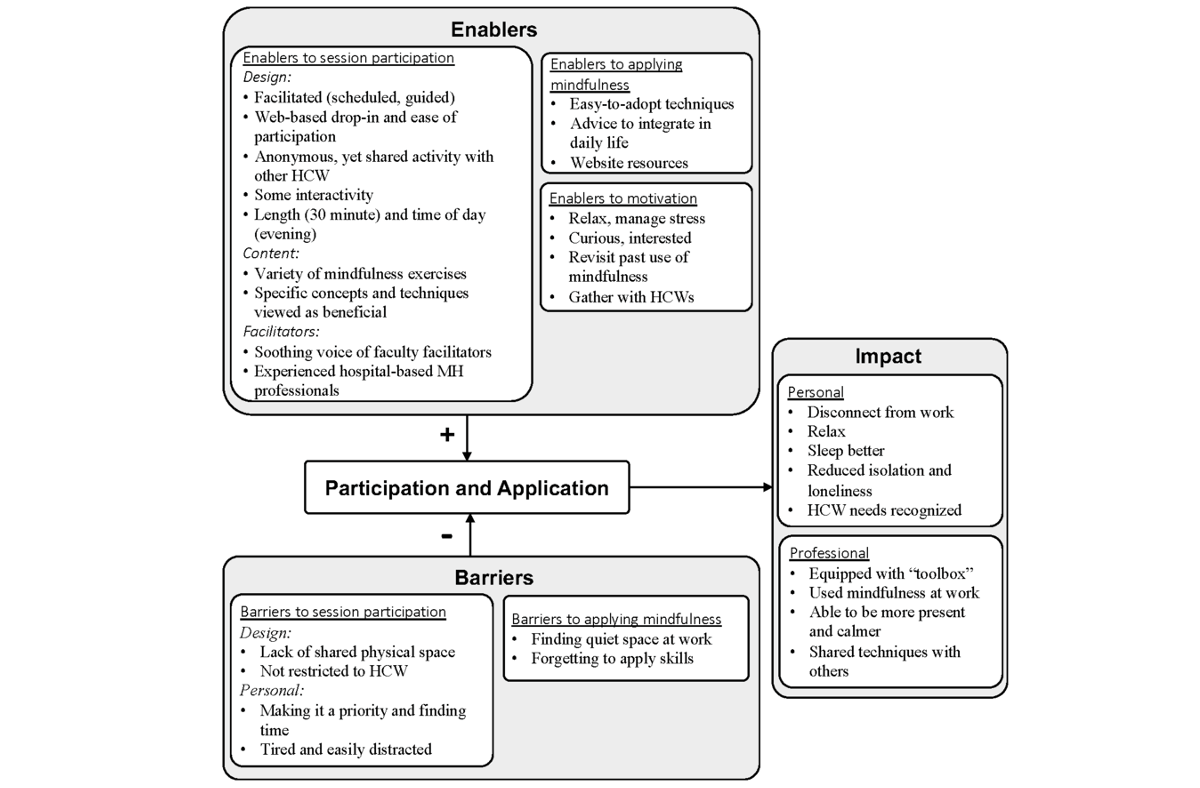
Framework of factors influencing the impact of web-based mindfulness. HCW: health care worker; MH: mental health.

Themes, experiences, enablers, and barriers to participation were related to respondents’ motivation, satisfaction, impact, and program design. Qualitative findings are described with select quotes and summarized below. Where participants’ quotes are used, we have specified their study number, role, age, number of sessions attended, whether they had applied the mindfulness skills taught, and the pandemic wave during which they were interviewed. A summary of findings is presented in Figure 3 and all data are included in Multimedia Appendix 5.

##### Motivation to Attend and Expectations

A variety of reasons motivated the participants to take part in the program. Most were looking for ways to relax and manage their stress:

Trying to help deal with the COVID and all the stress behind it. Dealing with the patients and their anxiety with it. I was kind of trying to be more relaxed.

#02 administrative assistant, aged 40-59 years, ≤5 sessions, applied learning, techniques, or tools in daily life, wave 1

Some participants wanted to decrease their sense of isolation created by the pandemic and have a way to connect with other HCWs:

I had been looking all over the place just for some kind of event or experience or connection of some kind for healthcare staff, healthcare teams because I felt really isolated.

#46 psychotherapist, aged 20-39 years, ≤5 sessions, applied learning, techniques, or tools in daily life, wave 2

Others said that they were curious about mindfulness, had been exposed to it in the past, saw this as an opportunity to pick it up again, and desired to gather with others and be a role model to encourage other staff to participate:

I am in a leadership position at the hospital and it was passed on to me as something that we could share with our staff. I wanted to encourage them to take it so I thought I should take it myself.

#14 manager, aged 40-59 years, ≤5 sessions, not applied learning, techniques, or tools in daily life, wave 1

When asked about expectations, participants largely said that they had none and came with an open mind. Several participants anticipated that it was likely to be beneficial but did not specify how:

I didn’t honestly think about it that much other than I know, at least on some level, that mindfulness practice would be good for me, that just by showing up it would be a positive thing.

#10, physiotherapist, aged 20-39 years, 6-10 sessions, applied learning, techniques, or tools in daily life, wave 1

Feeling quite unmotivated, just finding things at work difficult for the winter and the early part of spring, so it wasn’t necessarily fatigue, it was more feeling a bit overwhelmed with work.

#75, physician, aged 40-59 years, 6-10 sessions, applied learning, techniques, or tools in daily life, wave 3

##### Satisfaction

All participants said that the sessions were helpful, and some said that they surpassed their expectations and felt it was essential for getting through the pandemic:

It was more than what I expected. I thought I would try, to help out with the burnout and with the pandemic. But it was a lot more relaxing than I thought it would be. I was able to get into a really deeply relaxed state.

#22, psychotherapist, aged 20-39 years, ≤5 sessions, applied learning, techniques, or tools in daily life, wave 2

In order to keep working and go on in my life, it’s been one of the very important things for me.

#35, physician, aged 40-59 years, applied learning, techniques, or tools in daily life, wave 3, did not indicate the number of sessions they attended

When asked to rate the importance of the program to them on a scale from 1 to 10, in which 10 was very important, most participants articulated a moderately high score (mean 7.7, SD 1.48; median 8.0, range 5.0-10.0). Some participants commented on how this program was one of the several resources or supports, such as family, and that they were new to the program and could not yet fully appreciate how important the sessions may be:

I needed multiple things to help me through this time and so it was very important to me to have that regular time twice a week, where I would check-in and do these techniques to make sense of what’s going on, being able to deal with the difficult situation. In order to keep working and go on in my life, it’s been one of the very important things for me.

#35, physician, aged 40-59 years, applied learning, techniques, or tools in daily life, wave 3, did not indicate the number of sessions they attended; level of importance in life: score=10

I’ve barely scratched the surface... it’s the beginning of a learning. I’m hoping that if and when it ends, I will be able to continue this. A 10 out of 10 would be a sort of life-changing, I don’t think it’s life-changing. I would call it life-enhancing.

#78, physician, aged 40-59 years, >11 sessions, applied learning, techniques, or tools in daily life, wave 3; level of importance in life: score=8

I can’t say yet. I think I’m still pretty new. I’ve only done two sessions. Yes and no. In the sense that I think it’s nice to have that put in to your schedule a little bit but I can’t say that it’s translated into anything else in my life yet.

#44, physician, aged 40-59 years, ≤5 sessions, not applied learning, techniques, or tools in daily life, wave 2; level of importance in life: score=5

##### Impact

The participants articulated the numerous positive benefits of the mindfulness program. Most participants said that it prompted them to disconnect from work responsibilities and take time for self-care. As a result, they were able to unwind and relax at the end of the day and sleep better:

It helps me to realize it’s not all about going, going, going and bringing home your work and thinking about it 24/7. I think it’s a good way to almost detach a little bit from that stuff.

#44, physician, aged 40-59 years, ≤5 sessions, not applied learning, techniques, or tools in daily life, wave 2

It’s definitely easier to get into that relaxed state to go to sleep... I definitely feel more relaxed those nights when we have the session.

#22, psychotherapist, aged 20-39 years, ≤5 sessions, applied learning, techniques, or tools in daily life, wave 2

Most participants said that participation in the program helped them to be more present and calm and to better manage stress or anxiety. Many participants applied mindfulness techniques in their daily lives, including at work:

Being present, being consistent and calm when you are working in very high stress environments...I did try that out when I was at work, and especially when you’re feeling overwhelmed or just tired. And so, I think just being able to use those skills can help you think more clearly, tackle things in a calmer manner.

#01, physician, aged 20-39 years, >11 sessions, applied learning, techniques, or tools in daily life, wave 1

I’m much more productive with my time...and when you start to get more things done you don’t stress as much about it. It’s still pretty intense and it’s been a year and half...it just made me more level and able to work.

#78, physician, aged 40-59 years, >11 sessions, applied learning, techniques, or tools in daily life, wave 3

I found it a very useful tool to use, in my personal toolbox, to help me be more grounded, to be more productive and actually slow down and listen to others.

#78, physician, aged 40-59 years, >11 sessions, applied learning, techniques, or tools in daily life, wave 3

Engaging in a shared activity with other HCWs reduced feelings of isolation and loneliness, with gratitude for acknowledging HCWs as those who needed help to combat the undue stress of the pandemic:

I didn’t know any of the other people but you still kind of felt, even if you don’t know them everyone is facing similar challenges and probably having similar worries. That was nice to sit together.

#01, physician, aged 20-39 years, ≤5 sessions, applied learning, techniques, or tools in daily life, wave 2

It’s really comforting to see the same people coming, even if I don’t see them, sometimes I see their comments...I always like the idea of a community so feeling included or part of the group, where I’m not lost in a big system, so that helps.

#35, physician, aged 40-59 years, applied learning, techniques, or tools in daily life, wave 3, did not indicate the number of sessions they attended

A few participants mentioned additional positive impacts, such as sharing mindfulness techniques with patients whom they thought might benefit.

##### Design

When asked about the program design and format, participants appreciated the web-based, drop-in nature requiring no advance registration, the flow and variety of activities during each session, and the interactive components. Participants also appreciated the soothing voices, reassurances and reminders offered by faculty facilitators, the timing of the evening sessions, and the short 30-minute session length:

I love that it’s drop-in, you don’t have to register or sign up or make a plan.

#04, nurse practitioner, aged 20-39 years, 6-10 sessions, applied learning, techniques, or tools in daily life, wave 1

I joined a couple sessions where it seemed to flow really nicely. I thought that there was kind of an initial activity and then a little interlude moment and then a second activity.

#04, nurse practitioner, aged 20-39 years, 6-10 sessions, applied learning, techniques, or tools in daily life, wave 1

I really also enjoyed that there was a little bit of dialogue at the end, and I thought that was so nice for other people to share their comments, to be able to share mine.

#14, manager, aged 40-59 years, ≤5 sessions, not applied learning, techniques, or tools in daily life, wave 1

Furthermore, the participants noted specific aspects of the content they found helpful. In most cases, this referred to mindfulness exercises or techniques, for example, breathing exercises or body scans. In other cases, this referred to a concept, such as the need for self-care:

Self-compassion practices are probably the ones that stick out most.

#10, physiotherapist, aged 20-39 years, 6-10 sessions, applied learning, techniques, or tools in daily life, wave 1

The whole practice was around being kind to yourself and kind to others even though we may differ in our opinions or views etcetera. [...] And that was really impactful given the social-political state the world is in right now, so much divide and social injustice and political divide. It was really refreshing to talk about community and generosity and focus on that, when we’re bombarded everyday with the news and tv about what’s different about us right now.

#22, psychotherapist, aged 20-39 years, ≤5 sessions, applied learning, techniques, or tools in daily life, wave 2

My personality is that I always helped other people, family. That’s me. That’s my job and that’s also my personality. I always put everybody in front of me instead of taking care of myself. Which I think I do have to change a little bit as I am getting older [...] if I am forced to get into a place where I am the center and not everybody else but just me only, I think that would help me tremendously.

#48, physiotherapist, aged 20-39 years, ≤5 sessions, not applied learning, techniques, or tools in daily life, wave 2

##### Enablers

Participants noted multiple factors that enabled or challenged participation in Pause-4-Providers and the application of mindfulness outside the scheduled sessions.

These included a well-designed website and facilitated, web-based drop-in sessions. Although most participants appreciated the group nature of the sessions, they also valued their anonymity:

It’s convenient because it’s online, you don’t have to go somewhere, you can just Zoom in. It’s hard to get myself going somewhere to an actual place, but it is good to be with people. You kind of feel like you are with them, even if you don’t see them, so for me, the virtual format was perfect.

#35, physician, aged 40-59 years, applied learning, techniques, or tools in daily life, wave 3, did not indicate the number of sessions they attended

I think that’s a really powerful aspect of the program that invites more people to attend because you can change your screen name and be anonymous, so I thought that was cool. I think that anonymity aspect is really important so that everybody can just feel safe to access support and not have to reveal their identity from fear of judgement from coworkers.

#76, administrative assistant, aged 20-39 years, ≤5 sessions, applied learning, techniques, or tools in daily life, wave 3

Enablers to adopt or use mindfulness included faculty facilitators giving advice and examples on how to integrate practice into daily life, demonstrating techniques that were easy to adopt, and offering self-directed resources on the website to enable practice outside the sessions:

The way the facilitators are able to link the practices to real, realistic, day-to-day moments where the skills might come in handy [...] the suggestions, gentle reminders of where these skills can be useful in daily life.

#10, physiotherapist, aged 20-39 years, 6-10 sessions, applied learning, techniques, or tools in daily life, wave 1

Even throughout the day I think of some of the exercises. I’m seeing that start to change in a positive way. I’m taking a break from work at my desk, I stand up and I’ll take a moment with the practices in mind.

#46, psychotherapist, aged 20-39 years, ≤5 sessions, applied learning, techniques, or tools in daily life, wave 2

##### Barriers

Barriers to participation included competing demands that made it difficult to be a priority, being tired and feeling easily distracted, difficulty finding a quiet and private space at home, internet connection issues, and being seen by others in a susceptible state:

I guess the time of day. Although it is very convenient for me to attend, I do have to make a choice to do. Either I fit in time with my husband, or I prep for tomorrow or clean up. It’s a choice. You have to make a choice to go cause there’s always something else that you could be doing.

#04, nurse practitioner, aged <40 years, 6-10 sessions, applied learning, techniques, or tools in daily life, wave 1

Finding a quiet space in the house. I have to be really intentional about that part of things. I really need to set that up. [they compare to in-person] But now, even if I get in the room, even if I close the door and set it up, there’s still this possibility of other sounds in the house or somebody coming in. I think that’s a barrier to full participation, a distraction.

#46, psychotherapist, aged <40 years, ≤5 sessions, applied learning, techniques, or tools in daily life, wave 2

Some of the participants expressed a concern about appearing susceptible:

The one thing did cross my mind, this is a time I was hoping to be in my pajamas, wash my face, maybe lying down, and I wondered am I going to have to be on camera? I don’t want other people to see me in this kind of vulnerable state but that was quickly cleared up at the beginning of the Zoom call.

#22, psychotherapist, aged <40 years, ≤5 sessions, applied learning, techniques, or tools in daily life, wave 2

Barriers to adopting, applying, or using mindfulness outside of the sessions included forgetting or feeling unable to invoke learned skills and finding a quiet place at work to practice mindfulness:

It hasn’t been something that I have been trained to do so it takes a lot more effort and add-on to implement these new learned behaviors.

#22, psychotherapist, aged <40 years, ≤5 sessions, applied learning, techniques, or tools in daily life, wave 2

Participants variably preferred some practices over others, with none of the different practices being universally favored or disliked by the respondents. Participants offered 2 suggestions for promoting and supporting the adoption of mindfulness: offering quiet spaces at work and providing or referring participants to other resources that support mindfulness practice. The sessions were open to all HCWs, and some saw this as a barrier to feeling comfortable, with several participants offering the opinion that to create a safe space, consideration should be given to restricting the sessions to licensed clinical health care providers:

If one of the goals of the intervention was more engagement or discussion, or people sharing their stories, keeping it to the regulated professionals might have been more of a safe space, I guess. Not knowing who was on the call. I think at times kind of kept me from speaking up or asking questions or participating in discussions or feedback. Whereas, if it was just my peers, then it may have felt more of a safer space to talk.

#04, nurse practitioner, aged 20-39 years, 6-10 sessions, applied learning, techniques, or tools in daily life, wave 1

## Discussion

### Principal Findings

The strain placed on HCWs was evident from the start of the COVID-19 pandemic and persisted as the pandemic endured. This newly conceived well-being support program, Pause-4-Providers, consisted of synchronous, web-based, brief, drop-in mindfulness sessions for HCWs. It was feasible to implement as evidenced by its rapid deployment early in the first phase of the pandemic, with good use and continued session attendance during the first 3 waves of the pandemic (total attendance=1333). This program reached a wide range of HCWs, including physicians, a group typically more reluctant to attend well-being sessions, and more so during the pandemic [57]. It was acceptable to participants, with a large majority endorsing overall satisfaction of 5 out of 5 (32/47, 68%) and 4 out of 5 (11/47, 23%).

The Pause-4-Providers program was intentionally planned for implementation for HCWs with competing demands, time pressures, and exhaustion during the COVID-19 pandemic. Participants corroborated that they valued the program content and format that did not require registration, took place on the internet in the evening, was a drop-in session, and was of short duration (30 min) with the option of anonymity (eg, cameras could be switched off). The nonprescriptive nature of drop-in sessions allowed participants the choice to attend any number of sessions, unlike other structured mindfulness programs. Consistent with mindfulness literature, we observed a potential dose effect [31]. Participants who attended ≥2 sessions reported more positive benefits in coping skills (*P*<.001) and reduced perceived stress (*P*=.008).

Half of the participants attending Pause-4-Providers had moderate to high levels of burnout, as measured by the Single-Item Burnout Measure (26/50, 52%), similar to what others had during the COVID-19 pandemic [56]. This highlights the fact that some participants were in need of well-being interventions. In other words, some may have been motivated to alleviate burnout symptoms, while others sought to cultivate their well-being. We observed that resilience and burnout coexisted in this sample of HCWs during the COVID-19 pandemic.

The Pause-4-Providers study participants scored moderately high overall on the Resilience at Work Scale, with high subscale scores of “building networks, interacting cooperatively, finding your calling, and living authentically.” This may reflect HCWs’ need to pull together teams, with a shared purpose and a “calling”—resiliency factors that are essential during a pandemic. There is no ceiling on resilience, which can continue to grow. However, participants endorsed difficulty with the “maintaining perspective” subscale, scoring 48.1 (difficulty <50). This subscale represents an ability for flexible thinking and optimism. The pandemic has challenged HCWs to be adaptable and maintain positivity. The use of mindfulness training can help stressed individuals maintain perspective and shift from primitive survival stress responses (eg, fight, flight, freeze, or fold) to adaptive coping with mindful and reflective problem solving skills. The participants scored moderately on the subscale of “managing stress” aligned with their endorsed reasons for taking the course to relax and manage stress or anxiety. This is consistent with areas that mindfulness sessions can address by helping individuals to keep calm and reduce perceived stress [23,24,29-31]. These findings on the Resilience at Work measure were similar to those in a study by Maunder et al [56] with Canadian HCWs that evaluated the psychological impact of COVID-19.

The reported benefits of the program in the qualitative study included being better able to disconnect and decompress after work, seeing a need to prioritize self-care, and an improved ability to manage stress at work. This aligned with the survey findings of increased self-awareness of emotional states (25/45, 56%) and reduced stress (24/45, 53%). By providing a toolbox of mindfulness strategies and skills, participants were empowered to be calmer and more present. Brief mindfulness micropractices provided a potential mechanism for HCWs to navigate stressful and demanding workplace environments, as demonstrated in previous work done by one of the authors (ME) [13,35,36]. The themed agendas of each session focused on workplace applications of mindfulness practices. Respondents described how the particular use of micropractices allowed them to be more present, calmer, and more balanced at work. Some participants shared these mindfulness tools with their coworkers and patients. Participants also valued the faculty facilitators’ encouragement, tips, and examples of ways to use practices in the workplace, on the go.

Many participants did not turn on their cameras, although many did offer their thoughts and feedback (often gratitude) in the chat at the end of each session. Without their cameras turned on and in their homes in the evenings, the participants reported feeling less susceptible and more relaxed. Despite the anonymity of participation, the attendees endorsed a decreased sense of isolation in the context of the pandemic and felt connected to the group. We speculate that the combination of the live format, the commonality of being with HCWs during the pandemic, and the shared experience of practicing self-care in a group setting was sufficient to promote a sense of connection. This is similar to other mindfulness skills–based groups in which participants do not talk about their life experiences and yet endorse an increased sense of connectedness [37].

The use of an evolving curriculum allowed the team of faculty facilitators to perform practical ongoing needs assessment by checking in with attendees at the start of each session and flexibly adapting the session in response to the relevant needs of the attendees [36]. This was important as HCWs’ needs changed with the course of the pandemic. In the first wave, HCWs were frightened and needed to find ways to cope with uncertainty and change, whereas, with time, participants shared experiences of moral distress, exhaustion, and elements of posttraumatic stress [6,22]. The faculty facilitators could garner an iterative understanding of challenges and burnout factors with time as mental health experts, mindfulness specialists, and HCWs.

### Limitations

We largely relied on a top-down approach to invite HCWs to Pause-4-Providers sessions, contacting leadership in a wide array of institutions and health care settings. This dissemination strategy included outreach to interprofessional stakeholder organizations at the provincial, university, and hospital health care system levels. We did not track how or if they disseminated the information, and we did not have a consistent strategy to follow up with these organizations. Although this session recruitment strategy supported an expedient deployment strategy, it may have affected attendee recruitment.

Prioritizing anonymity and open access to drop-in web-based sessions with no required registration meant that we were not able to provide attendance reminders and relied on participant engagement and institutions to notify and remind potential attendees. This likely limited session attendance, retention, and study recruitment. Nonetheless, the study design was pragmatic for the drop-in nature of the program, with rolling recruitment during the first 3 waves of the pandemic. Approximately half (24/50, 48%) of the participants attended >2 sessions at the time of survey completion. Participants’ reports on resilience and burnout could have been affected by how many sessions they had attended before study enrollment and how far into the pandemic they started to attend sessions. We do not know if the consenting study participants were representative of the entire Pause-4-Providers attendee population or the HCWs’ population at large, limiting generalizability.

When the program was developed, we did not expect the pandemic to endure and have multiple waves. Attendance in the program was well distributed across the first 3 waves of the pandemic, and survey responses were concentrated between the end of wave 1 and the end of wave 2. It is possible that HCWs became more accustomed to the COVID-19 pandemic, with reduced stress and improved coping with time. Alternatively, the cumulative effect of the pandemic may have adversely affected the well-being of HCWs (eg, with increased levels of moral distress). The sample size was too small to determine how the motivations, benefits, satisfaction, and experiences of the participants may have differed across time points. Consistent with qualitative research standards, saturation of the findings was identified, and our sample size was consistent with the qualitative methodology [55,58].

It is unknown how representative the 50 study HCW participants were in relation to the larger sample of all attendees. The demographics did not include data on race, ethnicity, or intersectional identity, nor did they distinguish between work practice settings (eg, intensive care unit vs outpatient clinics) or whether HCWs were in COVID-19 patient-facing practice settings. Any of these factors could have affected or moderated the findings on burnout, workplace resilience, and pandemic-related stresses. The faculty facilitators in this project were White women psychiatrists. Future iterations of this program would benefit from a more culturally diverse and larger group of interprofessional faculty facilitators and participants. With respect to generalizability, qualitative findings were obtained from participants in the province of Ontario, Canada, and thus may not represent other health settings in other locations. Nurses represent approximately half of the health care workforce in Canada, and yet only 18% of the study participants were nurses. This low level of nurse participation may have been because of long, 12-hour shifts and competing priorities, such as family or childcare during the pandemic. This may also be related to free alternative support provided through their professional organizations (eg, individual psychotherapy) [6].

The format of live web-based mindfulness sessions is resource intensive as it relies on faculty facilitators; therefore, it may not be sustainable without funding or a dedicated team. Early in the COVID-19 pandemic, with heightened uncertainty and major changes in health care, faculty facilitated 8 evening sessions per week. As the pandemic continued, to reduce the burden on the faculty facilitators, the frequency of sessions was decreased to once a week.

### Conclusions

This descriptive, qualitative study and program evaluation demonstrated that the Pause-4-Providers program was feasible, acceptable, and appropriate, with high levels of satisfaction. Half of the participants endorsed moderate to high burnout, which may have motivated their attendance. Most participants endorsed positive benefits enabled by the curriculum, the faculty facilitators and the drop-in, low-dose, open-access format of the program. Importantly, participants reported feeling more relaxed after the sessions and using mindfulness tools at work and in their daily lives.

As Dana Faulds, an American poet, writes, “It only takes a reminder to breathe, a moment to be still, and just like that, something in me settles, softens, makes space for imperfection...I can make the choice to stop, to breathe, and be, and walk slowly into the mystery” [38]. The Pause-4-Providers program reminded HCWs to breathe, create space to be still, reflect on mindful self-compassion, and use microskills in the workplace and at home. To address the unprecedented negative psychological effects and burnout during the COVID-19 pandemic and support the well-being of HCWs beyond the pandemic, this program of brief web-based drop-in mindfulness merits further study.

## Appendix

Multimedia Appendix 1 Definitions of mindfulness concepts.

Multimedia Appendix 2 Pause-4-Providers questionnaire package.

Multimedia Appendix 3 Weekly number of attendees during the first 3 waves of the COVID-19 pandemic.

Multimedia Appendix 4 Pause-4-Providers qualitative interview guide.

Multimedia Appendix 5 Themes and exemplar quotes on motivation, expectations, design, and impact.

## Abbreviations

COREQ: Consolidated Criteria for Reporting Qualitative Research
HCW: health care worker

## Data Availability

The data sets generated and analyzed during this study are not publicly available because participants’ approval for public data sharing was not explicitly requested in the consent form approved by the research ethics board.
