# Supplementary material for: Novel Web-Based Drop-In Mindfulness Sessions (Pause-4-Providers) to Enhance Well-Being Among Health Care Workers During the COVID-19 Pandemic: Descriptive and Qualitative Study
Source: JMIR Form Res. 2024 Mar 14;8:e43875. doi: 10.2196/43875 (PMC10941832; doi:10.2196/43875)
Supplement: Multimedia Appendix 1 [file formative_v8i1e43875_app1.docx]

## Multimedia Appendix 1

### Definitions of mindfulness concepts

**Affectionate Breathing**

A meditation practice to ground in the present moment, by connecting with compassion and kindness to the calm and soothing rhythm of the breath [27].

**Three Minute Breathing Space**

A short and accessible meditation practice to respond skillfully to stressful situations [28]. Participants are invited to step out of automatic pilot and come into the present moment by focusing attention on the breath and sensations in the body.

**Loving Kindness Meditation**

A compassion-based, *metta* (ie. kindness, benevolence) meditation to cultivate self-healing and foster a deep sense of resilience. By expressing love and kindness towards oneself and others, participants expand the mind and cultivate increased focus and attention.

**Self-Compassion Break**

A soothing meditation practice to foster a sense of belonging and interconnectedness [27]. Participants are invited to move away from harsh self-criticism and welcome difficult experiences with self-compassion.
