# Supplementary material for: Novel Web-Based Drop-In Mindfulness Sessions (Pause-4-Providers) to Enhance Well-Being Among Health Care Workers During the COVID-19 Pandemic: Descriptive and Qualitative Study
Source: JMIR Form Res. 2024 Mar 14;8:e43875. doi: 10.2196/43875 (PMC10941832; doi:10.2196/43875)
Supplement: Multimedia Appendix 2 [file formative_v8i1e43875_app2.docx]

## Multimedia Appendix 2

### Pause-4-Providers questionnaire package

## Introduction

Thank you for considering filling out this brief 10-minute survey to evaluate the reach and impact of this drop-in, virtual mindfulness group whose goals are to reduce stress levels and enhance resiliency in the context of the COVID-19 pandemic. We gratefully acknowledge the support of the Ontario Ministry of Health's AFP Innovation Fund.

SAVE AND RETURN: You can save and return to the questionnaire at any time if you cannot complete it in one sitting. At the bottom of the page, there is a "save and return" button. When you click this, you will be provided with a return code which you can use at a later date to return to your questionnaire. Once you are ready to return, click the "returning" button on the top right-hand side of the page and input your return code. If you have any questions about this, please contact us.

**Consent Information**

**Participation is voluntary**

Your participation in the Pause 4 Providers research study is completely voluntary. You are welcome to complete as much or as little of the study as you please. You are allowed to skip questions if you don’t want to answer them. You may choose to withdraw from this research study at any point in time, even after you have provided consent. If you request to withdraw from this research study, please let us know via email. The research staff will acknowledge your decision and enact one of the following procedures to handle your data, based on your preference:

1. You will not be contacted for additional data collection. Any data collected from you will be immediately destroyed and excluded from the analyses.
2. You will not be contacted for additional data collection, but the research team may continue to use the data you provided before your withdrawal.

We will email you thanking you for your participation and confirming that you have been removed from the research study. Withdrawal from the study will not affect your participation in the Pause 4 Providers program. There are no professional consequences of this research study and this will in no way impact your position at your institution.

**Potential Risks and Benefits**

The risks we know of are minimal: There is a small risk of temporary emotional discomfort that may arise from being asked questions on the questionnaire, such as what drew you to participate in the sessions. You can choose not to answer any questions that you are not comfortable answering.

You will not receive direct benefit from your participation in this research study. Information learned from this research study may help to develop effective mindfulness programs in the future.

**Confidentiality**

Your confidentiality is important to us. The information that you provide will be stored within the survey system databases and downloaded to be stored on servers at Mount Sinai Hospital in Toronto, Canada. Only the study team will be allowed to look at your anonymized records. The overall results of this research may be published in a medical or scientific journal, or presented at a medical or scientific meeting. Individual answers will not be published, displayed or discussed.

If you choose to participate in the qualitative interview, the research team will collect your email address. This information will be stored separately from the rest of the information collected for this program evaluation (called 'coded data'). The research team will have a list that links your email address to your code so that your coded data can be linked back to you if necessary. This list will be kept separate from the coded data in a secure place. If the results of this study are published, any identifiable information will remain confidential. Even though the likelihood that someone may identify you from the coded data is very small, the risk can never be completely eliminated.

Information that travels on the internet and is stored on a computer is sometimes viewed by third parties by accident, because of government policies or a court subpoena, as a result of criminal hacking or for other reasons. Therefore, completing this questionnaire carries some risk of your personal, anonymous information being read by someone other than the researchers.

**Approval by the Mount Sinai Research Ethics Board**

This research study has been approved by the Mount Sinai Hospital Research Ethics Board. If you have any questions, concerns or would like to speak to the study team for any reason, please contact Camille Khallouf at camille.khallouf@sinaihealth.ca. If you have any questions about your rights as a research participant or have concerns about this study, please contact the Chair of the Mount Sinai Hospital Research Ethics Board or the Research Ethics Office at 416-586-4875. The REB is a group of people who oversee the ethical conduct of research studies. These people are not part of the study team. Everything that you discuss will be kept confidential.

By completing the questionnaire, you agree that:

- You voluntarily agree to participate in the Pause 4 Providers research study and have your anonymized responses included in the evaluation.
- You have been given time to consider whether or not to participate in the Pause 4 Providers research study.
- You understand that you may refuse to participate or complete as much or as little of the questionnaire as you please without any adverse or negative consequences.
- You understand that you may refuse to participate or withdraw from this research study at any time and that this withdrawal will not have any adverse or negative consequences or affect any future involvement with the Pause 4 Providers program.
- You understand that your implied consent does not waive any of your legal rights, nor relieve the Pause 4 Providers team from any legal responsibility.
- You have read and understood this entire consent form.

1. **Please choose one: ***

☐ I consent to the Pause 4 Providers research study.

☐ I do not consent to the Pause 4 Providers research study and wish to exit.

1. **Have you completed this survey before? ***

☐ Yes, I have completed the survey before.

(Thank you for your help and participation! We are only asking participants to fill out this questionnaire once.)

☐ No, this is my first time completing this survey.

**Demographic Information**

1. **What is your primary profession?** Select which best applies.

☐ Administrative/Education support

☐ Behaviour Analyst

☐ Case worker

☐ MD (Family Physician)

☐ MD (Specialist)

☐ Nurse Practitioner

☐ Occupational Therapist

☐ Pharmacist

☐ Physician Assistant

☐ Physiotherapist

☐ Psychologist

☐ Psychotherapist

☐ Registered Nurse

☐ Resident

☐ Social Worker

☐ Other

1. **Gender identity:**

☐ Female

☐ Male

☐ Intersex

☐ Trans-Female to Male

☐ Trans-Male to Female

☐ Two-Spirit

☐ I don't know

☐ Prefer not to say

☐ None of the above

1. **How old are you?**

☐ 19 or under

☐ 20-39

☐ 40-59

☐ 60 or older

1. **What province are you from?** (If from other an country than Canada, please specify)

☐ Ontario

☐ Québec

☐ Nova Scotia

☐ New Brunswick

☐ Manitoba

☐ British Columbia

☐ Prince Edward Island

☐ Saskatchewan
 ☐ Alberta

☐ Newfoundland and Labrador

☐ Other:

|  |
| --- |

#### **What is your primary practice setting?**

#### ☐ Community clinic

☐ Out-patient hospital service

☐ ICU

☐ Other

☐ Prefer not to say

1. How did you hear about Pause 4 Providers?

#### ☐ Hospital Newsletter

#### ☐ Facebook

#### ☐ Instagram

#### ☐ Twitter

#### ☐ Recommended from a peer

#### ☐ Professional Organisation

#### ☐ Virtual Flyer

☐ Lecture

☐ Other

|  |
| --- |

**Single-Item Burnout Measure (Dolan et al, 2015)**

1. **Overall, based on your definition of burnout, how would you rate your level of burnout?**

☐ I enjoy my work. I have no symptoms of burnout.

☐ Occasionally I am under stress, and I don’t always have as much energy as I once did, but I don’t feel burned out.

☐ I am definitely burning out and have one or more symptoms of burnout, such as physical and emotional exhaustion.

☐ The symptoms of burnout that I’m experiencing won’t go away. I think about frustration at work a lot.

☐ I feel completely burned out and often wonder if I can go on. I am at the point where I may need some changes or may need to seek some sort of help.

**Resilience at Work Scale (25 item scale)**

|  | **Strongly Disagree** | **Disagree** | **Somewhat Disagree** | **Neither Agree nor Disagree** | **Somewhat Agree** | **Agree** | **Strongly Agree** |
| --- | --- | --- | --- | --- | --- | --- | --- |
| 10. I have developed some reliable ways to deal with the personal stress of challenging events at work. |  |  |  |  |  |  |  |
| 11. My personal support network is important to my coping at work. |  |  |  |  |  |  |  |
| 12. Nothing at work ever ‘fazes me’ for long. |  |  |  |  |  |  |  |
| 13. I'm not afraid to seek advice and support if and when I need help with my work. |  |  |  |  |  |  |  |
| 14. I have important core values that I hold fast to in my work-life. |  |  |  |  |  |  |  |
| 15. I am careful to maintain a good level of physical fitness. |  |  |  |  |  |  |  |
| 16. My work place is somewhere where I feel that I belong. |  |  |  |  |  |  |  |
| 17. Negative people at work tend to pull me down. |  |  |  |  |  |  |  |
| 18. I know my personal strengths and make sure I use them regularly in my work. |  |  |  |  |  |  |  |
| 19. I believe in giving help to my work colleagues, as well as asking for it. |  |  |  |  |  |  |  |
| 20. I have friends at work whom I can rely on to support me when I need it. |  |  |  |  |  |  |  |
| 21. The work that I do helps to fulfil my sense of purpose in life. |  |  |  |  |  |  |  |
| 22. I am able to change my mood at work when I need to. |  |  |  |  |  |  |  |
| 23. The work that I do fits well with my personal values and beliefs. |  |  |  |  |  |  |  |
| 24. When problems arise at work I focus on finding a solution rather than just worrying about them. |  |  |  |  |  |  |  |
| 25. When things go wrong at work, it usually tends to overshadow the other parts of my life. |  |  |  |  |  |  |  |
| 26. Keeping physically fit helps me cope with the demands of my work. |  |  |  |  |  |  |  |
| 27. I make sure I take breaks to maintain my strength and energy when I’m working hard. |  |  |  |  |  |  |  |
| 28. Generally I appreciate what I have in my work environment. |  |  |  |  |  |  |  |
| 29. I have developed some reliable ways to relax when I’m under pressure at work. |  |  |  |  |  |  |  |
| 30. I have a strong and reliable network of supportive colleagues at work. |  |  |  |  |  |  |  |
| 31. I often ask for feedback so that I can improve my work performance. |  |  |  |  |  |  |  |
| 32. I am careful about eating well and healthily. |  |  |  |  |  |  |  |
| 33. I am careful to ensure that my work does not dominate my personal life. |  |  |  |  |  |  |  |
| 34. I know myself and my feelings honestly and realistically. |  |  |  |  |  |  |  |

**Feedback Questionnaire**

#### **35. How many drop-in mindfulness sessions have you attended so far?** (please choose the best answer from the ones below)

☐ 1

☐ 2-5

☐ 6-10

☐ 11 or more

#### **What motivated you to attend the sessions?**

#### Here are some examples that might motivate people to attend sessions. Please indicate your level of motivation on each of the examples below.

|  | **Not at all** | **A bit** | **Moderately** | **Very much so** | **Definitely** |
| --- | --- | --- | --- | --- | --- |
| 36. To feel less isolated |  |  |  |  |  |
| 37. To help with emotional reactivity |  |  |  |  |  |
| 38. To cope at home |  |  |  |  |  |
| 39. To relax |  |  |  |  |  |
| 40. To improve sleep |  |  |  |  |  |
| 41. Wish for loving kindness/ self-compassion |  |  |  |  |  |
| 42. Manage stress or anxiety |  |  |  |  |  |
| 43. To give me energy |  |  |  |  |  |
| 44. Tips to cope with work |  |  |  |  |  |
| 45. To be in a community |  |  |  |  |  |
| 46. To learn mindfulness |  |  |  |  |  |

# 47. Other reasons for attending (please specify):

|  |
| --- |

#### **48. In the context of the COVID-19 pandemic, compared to when you were not attending the P4P session(s), in what ways are you feeling or doing/coping differently -- better, worse, or unchanged; and what do you attribute this to?**

|  |
| --- |

#### **To what degree have you experienced the following as a result of attending the virtual mindfulness sessions?**

|  | **Not at all** | **A bit** | **Somewhat** | **Very much so** | **Significantly, a lot** |
| --- | --- | --- | --- | --- | --- |
| 49. Enhanced your coping skills |  |  |  |  |  |
| 50. Reduced your stress level |  |  |  |  |  |
| 51. Improved your capacity to work with your teams day-to-day |  |  |  |  |  |
| 52. Increased your self-awareness of emotional states |  |  |  |  |  |
| 53. Enhanced your ability to be responsive rather than reactive to challenges as they arise |  |  |  |  |  |

#### **54. What impact has COVID had on your role and activity in Spring/Summer 2020 and what do you foresee in the coming months?​**

|  |
| --- |

#### **55. Please indicate your level of satisfaction with the virtual mindfulness sessions.**

1 2 3 4 5

**56. Are you willing to be contacted by us to participate in an interview to gather more qualitative information?** If yes, please provide your email address.

|  |
| --- |

Thank you for your help and participation! Your input is essential for the development of our program and we truly appreciate your valuable feedback.

Please use the space below if you would like to provide any feedback on the questionnaire, or if you have any other comments or questions.

|  |
| --- |
