# Supplementary figures and images for: Novel Web-Based Drop-In Mindfulness Sessions (Pause-4-Providers) to Enhance Well-Being Among Health Care Workers During the COVID-19 Pandemic: Descriptive and Qualitative Study
Source: JMIR Form Res. 2024 Mar 14;8:e43875. doi: 10.2196/43875 (PMC10941832; doi:10.2196/43875)

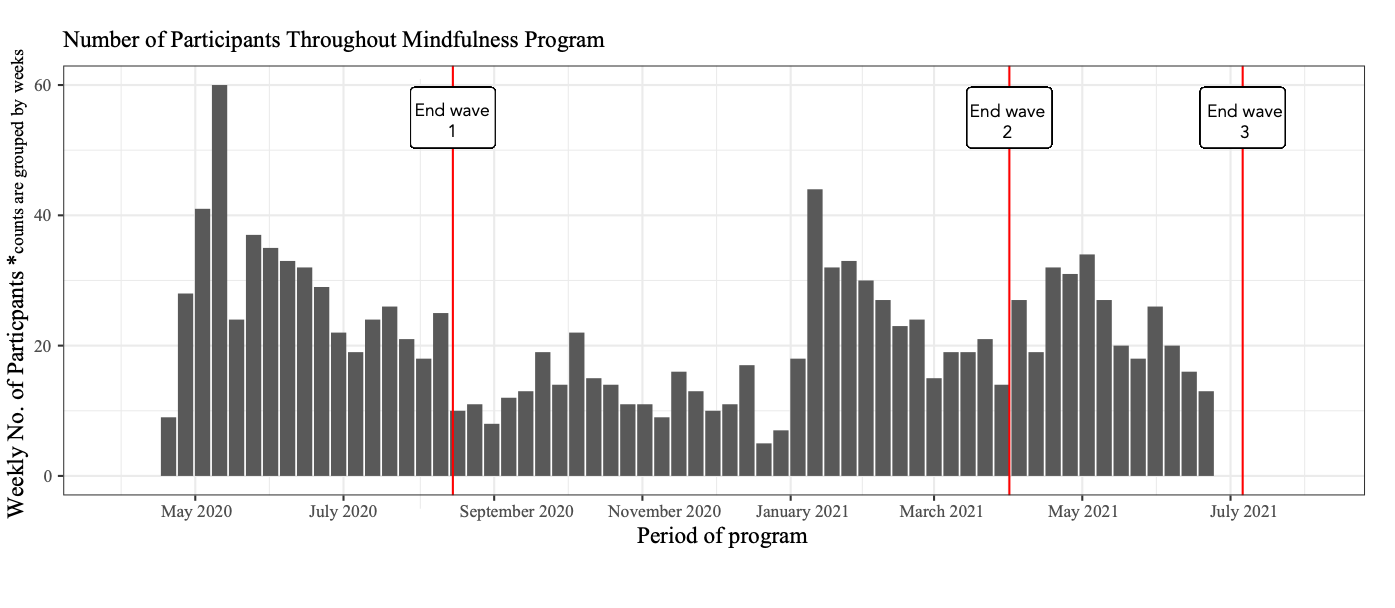

Supplement: Multimedia Appendix 3 [file formative_v8i1e43875_app3.png]
