# Supplementary material for: Novel Web-Based Drop-In Mindfulness Sessions (Pause-4-Providers) to Enhance Well-Being Among Health Care Workers During the COVID-19 Pandemic: Descriptive and Qualitative Study
Source: JMIR Form Res. 2024 Mar 14;8:e43875. doi: 10.2196/43875 (PMC10941832; doi:10.2196/43875)
Supplement: Multimedia Appendix 4 [file formative_v8i1e43875_app4.docx]

## Multimedia Appendix 4

Pause-4-Providers qualitative interview guide.

**INTRO**

- Thanks so much for making the time to speak with me today
- This interview is being conducted as part of a research study to assess participant views about the design and impact of the Pause 4 Providers program.
- We will use feedback to refine the program
- I will ask you 6 main questions. The interview requires about 20 minutes
- Please respond with as much detail as you can provide
- You may choose not to answer certain questions, and you can stop the interview at any time
- Before we begin, do you have any questions?

**QUESTIONS**

**Expectations**

| Main questions | Additional probes if needed  Follow-up with ideas they share, which may not match these probes; use these probes judiciously if they provide little response or detail |
| --- | --- |
| What prior mindfulness experience did you have? | *This is for demographic information* |
| What motivated you to participate in Pause-4-Providers? | Why were you interested in or curious about mindfulness in particular versus another form of relaxation?  What motivated you to join? (i.e. helpful in general, easy to join, anonymous, address isolation/loneliness; feeling anxious, stressed, depressed; looking for a way to relax at end of day) |
| Before it began, did you have any expectations, hesitations or concerns about the program? | The format or content  Anonymity  Whether mindfulness would help you  Whether you would be able to learn it or practice it  Whether you could find time |

**Impact**

| Main questions | Additional probes if needed  Follow-up with ideas they share, which may not match these probes; use these probes judiciously if they provide little response or detail |
| --- | --- |
| Was the program what you expected? | They were vague when answering this question. They said it met their expectations or exceeded their expectations.  In what way did it meet your expectations? (e.g. content, format, benefits)  Was there anything about it that surprised you? |
| How did the program benefit you? | How did it help you in your professional work?  How did it help you as a person?  Did you develop knowledge about mindfulness?  Did you learn about different techniques or exercise you can employ?  Did you learn something about yourself?  Do you feel better prepared to manage stressful situations in the future?  Another other learning or impact?  If they say “too soon to know” because they only did one or a few sessions, ask something like: based on the X sessions that you did, do you have a sense of how it might help you? |
| Do you use or apply any exercises or techniques that you learned? | Which ones?  Why those exercises or techniques?  In what situations do you use them?  How often do you use them?  In those situations, how do they help you? |
| How important to you is Pause-4-Providers on a scale from 1 to 10 where 10 is very important? | Please explain your rating |
| Would you recommend it to others? | What about it would you recommend? |

**Design**

| Main questions | Additional probes if needed  Follow-up with ideas they share, which may not match these probes; use these probes judiciously if they provide little response or detail |
| --- | --- |
| Content:  What techniques or exercises did you like? | What did you like about them?  In what way were they helpful to you?  What did you not like and why?  Was there anything you learned that was helpful? |
| Format:  What about the format made it easy to participate?  How well did the virtual format work for you? | Was it easy to register?  Was it easy to connect to each session?  Was it easy to follow instruction and do the exercises given the virtual format? |
| Personnel:  What about the facilitators did you like? | Techniques they used, their voice, the way they provided instructions?  What else would you like facilitators to do or teach you? |
| Timing:  How well did the time of day suit you, referring to 8:30 pm in the evening?  How well did session length suit you, referring to 30 minute sessions?  How well does the frequency suit you, referring to twice per week? | What time would suit you better and why?  What session length would suit your better and why?  How often would like sessions to be? |

**Determinants**

| Main questions | Additional probes if needed  Follow-up with ideas they share, which may not match these probes; use these probes judiciously if they provide little response or detail |
| --- | --- |
| Enablers:  What factors promoted or supported or helped you to participate?  What factors support or help you to practice mindfulness outside of the sessions? | Virtual  Scheduled time  Drop-in format  Personality/instructions of facilitator  Shared experience with other healthcare professionals  Specific examples of how to incorporate in daily life |
| Barriers:  What factors were barriers of participating?  What factors are barriers of you practicing mindfulness outside of the sessions? | Finding time  Remembering to do it  Remembering the exercises/techniques  Competing priorities  Making it a priority  Focusing  Setting (intrusions at work or home)  Lack of space in which to practice  Can’t do it without guidance of a facilitator |

**Recommendations**

| Main questions | Additional probes if needed  Follow-up with ideas they share, which may not match these probes; use these probes judiciously if they provide little response or detail |
| --- | --- |
| How can we improve the program? | Additional resources to support practice outside of sessions  More sessions/sessions at different times  Different techniques or exercises  reminders |
