# Supplementary material for: Novel Web-Based Drop-In Mindfulness Sessions (Pause-4-Providers) to Enhance Well-Being Among Health Care Workers During the COVID-19 Pandemic: Descriptive and Qualitative Study
Source: JMIR Form Res. 2024 Mar 14;8:e43875. doi: 10.2196/43875 (PMC10941832; doi:10.2196/43875)
Supplement: Multimedia Appendix 5 [file formative_v8i1e43875_app5.docx]

**Multimedia Appendix 5. Themes and exemplar quotes on motivation, expectations, design, and impact.**

**Motivation and Expectations**

| Theme | Age <40 | | Age 40-60 | |
| --- | --- | --- | --- | --- |
|  | Clinicians (physicians, nurse practitioners) | Allied health and other (physiotherapist, social worker, psychotherapist, admin asst, manager) | Clinicians (physicians, nurse practitioners) | Allied health and other (physiotherapist, social worker, psychotherapist, admin asst, manager) |
| Relax and manage stress |  | I was starting to feel pretty burned out before the pandemic, and then the pandemic happened and it started to feel unmanageable. I signed up for that workshop just for the credits, and when I heard about Pause 4 Providers, I was like, oh my god I definitely need to do this (22 psychotherapist age <40, <5 sessions, applied, wave 2)  At the very least I was hoping to just feel a little lighter at the end of my day, a little bit more relaxed, not as buzzing. I was starting to have a hard time with my sleep and my appetite given how burned out I was feeling. I thought that given the time slot at 8.30pm, that was pretty ideal to get my body in more of a ready state for sleep. Versus, just trying to recover from my day and my shift and then come home, watch something, eat something, read something and go to sleep. I didn’t really have that protected unwinding space (22 psychotherapist age <40, <5 sessions, applied, wave 2)  Basically life started feeling really busy during the lockdowns and I wanted to intentionally create space in my schedule to have time for myself, so that was the main reason (76 admin assist, age <40, # sessions, applied, wave 3)  I thought that it would help me feel more relaxed in my everyday life, to not take things as seriously, to give me also space to deal with my feelings, you know sadness, grief, anger all those sorts of emotions, to kind of process them (76 admin assist, age <40, # sessions, applied, wave 3) | In general, everything has been more stressful. Work related was just more confusing. You’re not sure what to do, protocols change all the time. In general, there were so many changes. And patients were scared, they didn’t want to come in. And you’re trying to get your job done and patients end up not showing up (44 physician age 40-60, <5 sessions, not applied, wave 2)  I was looking for something to support me because I was going through stress…This whole change with the COVID…Very stressful events that happened in the beginning of the COVID and the intubation and stuff…I felt like I needed some mindfulness to help me deal with those things (35 physician age 40-60, #sessions, applied, wave 3)  Feeling less anxious, maybe feeling like I could attend to my day in a more mindful way (75 physician age 40-60, # sessions, applied, wave 3)  I just needed a way to take my mind off everything that was going on at once and just to settle it down. You know, it felt like whack-a-mole and I was just looking for something to help me focus on the issues at hand with all of the noise and distractions (78 physician age 40-60 #sessions applied wave 3)  I was just having a hard time being focused. And it’s pretty stressful and I was bringing it all home, even the stuff that I couldn’t control myself. And, you know, having a hard time getting away from feeling bad that I couldn’t solve issues that I was not able to solve by myself. It’s been pretty intense. Ramp up, ramp down, ramp up, having to spend all my time working with my teams to plan. So it’s been a pretty intense year and a half (78 physician age 40-60 #sessions applied wave 3) | I started participating in it because sometimes during COVID doing the mindfulness in the evening kind of helped shut my mind off so I can try to relax and go to sleep, instead of my mind keep racing with thoughts (02 admin asst age 40-60, <5 sessions, applied, wave 1)  Trying to help deal with the COVID and all the stress behind it. Dealing with the patients and their anxiety with it. That’s what, I was kind of trying to be more relaxed myself and not get my knickers in the knot shall we say (02 admin asst age 40-60, <5 sessions, applied, wave 1)  I enjoy mindfulness. And with the unpredictabilities of the earlier stages of the pandemic stressful, mindfulness helps feel more grounded. And you know just allows for a better lifestyle (06 social worker age 40-60, 10+ sessions, applied, wave 1)  I hoped it would allow for that half hour at the end of the day for grounding, to really decompress (06 social worker age 40-60, 10+ sessions, applied, wave 1)  Also feeling the stress that everybody else is from COVID and thought it would be a good thing to try (14 manager age 40-60, <5 sessions, not applied, wave 1)  Because of COVID, it was a very stressful time, so it was perfect timing for this to come out (79 other age 40-60 applied wave 3)  Last spring, there was so many unknowns and we had no idea what we were dealing with. We didn’t have enough PPE, we were being put in an unsafe position from a work perspective, you know, being told that this was “quote unquote” safe, even though nobody really knew, right? So that was incredibly stressful (79 other age 40-60 applied wave 3) |
| Curious, interested or drawn to mindfulness | Initially it was curiosity… And then as it evolved, I enjoyed going to the sessions and I found that I slept much better that evening and I was looking for something to help me wind down at the end of the day (04 nurse practitioner age <40, 5-10 sessions, applied, wave 1) | I see myself as being deeply spiritual and I think the most empowering wellness techniques are the ones that teach us to rely on ourselves and use our own strengths and gifts, and so I think mindfulness is really perfect for that because it’s just about connecting to yourself, it’s not about anything external to you so it really gives you your power back (76 admin assist, age <40, # sessions, applied, wave 3) | It was just something that was forwarded. I was like yes that looks interesting, I’ll try (44 physician age 40-60, <5 sessions, not applied, wave 2)  it was forwarded to us by our chief so okay this must be… It was just something of interest. I think a colleague of mine also heard about it so I didn’t have any. It just sounded like it’s a great starting point. It looks like a good thing to try (44 physician age 40-60, <5 sessions, not applied, wave 2)  I’m curious…because in my job you need to have situational awareness, so I like the idea that you allow all the thoughts away… The mind-body connection, I like being in touch with how you are feeling your behavior (35 physician age 40-60, #sessions, applied, wave 3)  I tried going into it being sort of open minded and curious…and so I thought, let’s just give it a try (78 physician age 40-60 #sessions applied wave 3) | I was always to open those kinds of mindfulness. We received an email that there is an option to do that, so I wanted to try it out (48 physiotherapist age 40-60, <5 sessions, not applied, wave 2) |
| Opportunity to revisit past exposure to mindfulness | I have been doing things that are very self-directed. What is it like 10 percent? There are a few apps I signed up for. 10 percent which is a [27:30] meditation app and then I also did the Deepak Chopra 21-day meditation challenge or guided meditation. I’s 21 days of 13 minutes of meditation I also did. I didn’t finish it but there was this thing called the next 90 days which was through a coaching service called Hollis Company [28:00]. It was kind of cool. Every Monday they would release a topic like a lesson on a topic like organization or I don’t know, the importance of exercise. I don’t know some sort of lifestyle coaching thing. But actual formal therapy and one on one therapy, I’ve never done (04 nurse practitioner age <40, 5-10 sessions, applied, wave 1) | I’d had a bit of exposure to mindfulness probably 3 or 4 years ago, it was a nice opportunity to formally re-engage in some sort of structured way with a mindfulness practice (10 physiotherapist age <40, 5-10 sessions, applied, wave 1)  Mindfulness obviously it’s a practice that takes some commitment and takes a little bit of skill, or at least patience. Being something that I don’t have a lot of consistent practice with…I didn’t really hesitate but was sort of a mindful decision to make an effort to show up and participate (10 physiotherapist age <40, 5-10 sessions, applied, wave 1)  It has been really helpful as a reminder. It’s really helpful returning to practices that I completed ignored, that used to be a bigger part of my life (46 psychotherapist age <40, <5 sessions, applied, wave 2)  Last spring, I started doing mindfulness a little bit on my own and I would do that maybe a couple of times a week, it was all just self-directed learning (76 admin assist, age <40, # sessions, applied, wave 3) | Probably even pre-COVID I have always thought that I should do some form of therapy but never really jumped in to be honest. Yes, so probably it was almost. What the word I’m looking for. A fortunate time that it just kind of came up, it was easy to do, it was free, my friend told me I should do it and then it was right there. Yes, probably for the last maybe 3 or 4 years since the birth of my second child just thinking that it would serve me to spend more time on myself (04 nurse practitioner age <40, 5-10 sessions, applied, wave 1)  I’d taken a one-day maybe 5 or more years ago and I’d done a continuing education course through the city as well on mindfulness that was over 6 to 8 weeks like an evening type thing, and then I had done some readings and very intermittent practice (75 physician age 40-60, # sessions, applied, wave 3)  I have read a book. I practiced briefly, I gave it up (78 physician age 40-60 #sessions applied wave 3) | I’ve been doing it for over 5 years, so for continuation (79 other age 40-60 applied wave 3) |
| Opportunity to gather with other healthcare workers | I was expecting it to be geared towards healthcare professionals, the type of work that we are doing (04 nurse practitioner age <40, 5-10 sessions, applied, wave 1)  Usually when I meditate it’s not with other people so I thought this would maybe foster a little more of a community. And then also I guess the unique thing about this one is that it is for healthcare providers so would be fostering a sense of connection. Especially when it started it was just the beginning of this pandemic so a way for people to come together in that way 01 physician age <40, 10+ sessions, applied, wave 1)  It’s not expected to be this very interactive type of program so to speak. Just even the fact that you know other people are sitting and meditating at the exact same time and they might be in a different city, or not sure if there is anyone in other provinces, but even just that sense of connection virtually and then that layer of accountability. It’s at a set time, you know who is there, you know who is showing up, you feel like you are doing it together even though of course everyone is sort of in their own place (01 physician age <40, 10+ sessions, applied, wave 1) | I felt there were no other resources available for healthcare providers to gather together. I actually couldn’t find anything in terms of resource like that before I found Pause 4 Providers (46 psychotherapist age <40, <5 sessions, applied, wave 2)  I had been looking all over the place just for some kind of event or experience or connection of some kind for healthcare staff, healthcare teams because I felt really isolated. Just my workplace and my colleagues, I felt there was a lot of people that were not willing to share, and I wanted to see if there was any kind of space or opportunity to do that because I thought there might be more people like me (46 psychotherapist age <40, <5 sessions, applied, wave 2)  The [mindfulness] facilitators offered encouraging remarks around just people being there for one another and through creating a community of practice how that can be a form of support, even though we’re not necessarily showing our faces or talking directly to one another. That just sort of showing up, showing some sort of solidarity or you know caring for one another as people all participating or delivering healthcare at a difficult time (10 physiotherapist age <40, 5-10 sessions, applied, wave 1)  I was actually hoping to find a connection with the other providers. That was my hope. It was actually more like a community feel, more than the mindfulness piece (46 psychotherapist age <40, <5 sessions, applied, wave 2)  I just wanted to see in what way this could be some kind of a community, or even a brief connection, even though it’s kind of superficial, with other providers (46 psychotherapist age <40, <5 sessions, applied, wave 2)  I hope that it is not going to drop off because I don’t know what I would do actually [laugh]. I don’t know what I would find in place of this. I’ve put in a good amount of time searching and I couldn’t find anything that is specific for healthcare providers, which was the first thing. This whole no one understands me, which they literally don’t outside of our field. It isn’t their fault but it is just a unique experience. And especially in our university departments. It’s so huge, it’s so intense, it’s so overwhelming and so chaotic. It’s really unfair to impose that on other people. I think it just makes it all the more lonely. I think it’s a profound loneliness in all of this that I was hoping to address. I really found that this was a good fit for that, so I really hope they continue (46 psychotherapist age <40, <5 sessions, applied, wave 2)  Obviously I could of done mindfulness on my own but knowing that there was other people that were going to do it on Zoom at the same time was really motivating so it helped me take care of myself (76 admin assist, age <40, # sessions, applied, wave 3)  And I also thought that it would help me feel supported, you know just the idea to know that there are people out there who appreciate this, they are also seeking help for themselves and I am not alone. This is also a really important piece of it I think (76 admin assist, age <40, # sessions, applied, wave 3) | I thought it might help with mental health and coping, kind of a community, because I can go to work but I can’t go to other groups as much, so even though you don’t see the people [during sessions], you see, oh that name has been there, yes, kind of like a community (35 physician age 40-60, #sessions, applied, wave 3) | I liked the timing of it and I liked the community aspect of it, that it was being offered by somebody who was working in a similar field to me (14 manager age 40-60, <5 sessions, not applied, wave 1) |
| Role model self-care behaviour to other staff |  |  |  | I am in a leadership position at the hospital and it was passed on to me as something that we could share with our staff. I wanted to encourage them to take it so I thought I should take it myself (14 manager age 40-60, <5 sessions, not applied, wave 1) |
| Continuing education credit |  | I was just initially looking to get some extending education credits for my college. I am a registered psychotherapist. Every couple of years we need a certain amount of hours for continuing education credits. I thought that would be interesting (22 psychotherapist age <40, <5 sessions, applied, wave 2) |  |  |
| No advance expectations of P4P | I wasn’t really sure what to expect because there is a lot of different styles and types of meditation or mindfulness practices. I tried to just have an open mind…I wasn’t that familiar with Zoom in general, how it works and so it was figuring that out. I wasn’t sure everyone was going to have their camera on or not, and if people were going to be expected to talk at the end or something like that, if there was like a question period or something like that. I wasn’t really sure if it was going to be like that (01 physician age <40, 10+ sessions, applied, wave 1) | I didn’t really have any specific expectations. I knew that it was sort of a 30-minute regular guided mindfulness practice for healthcare providers with the lens of supporting one another and self-care in the midst of COVID related stresses. Other than that, I didn’t have any specific expectations (10 physiotherapist age <40, 5-10 sessions, applied, wave 1)  I tried not to have any (22 psychotherapist age <40, <5 sessions, applied, wave 2)  I was unsure what it would like but I thought it would include some kind of mindfulness component and also coming together as a group. I wasn’t sure in what way (46 psychotherapist age <40, <5 sessions, applied, wave 2) | I came with no expectations because I had no clue what to expect (44 physician age 40-60, <5 sessions, not applied, wave 2) | I didn’t really have any expectations. It’s something new to me. I had never done the mindfulness before so I didn’t know what it was about. There were really no expectations, it was basically just going in, trying it out and see what it was about. It was more curiosity (02 admin asst age 40-60, <5 sessions, applied, wave 1)  I didn’t really have any expectations…I was aware it was a half hour opportunity, four times a week to be involved with a guided mindfulness meditation exercise… and it was what I was looking for (06 social worker age 40-60, 10+ sessions, applied, wave 1)  I had none. I was totally open minded. I had no expectations. I did not know what it was about, so I really had no clue (48 physiotherapist age 40-60, <5 sessions, not applied, wave 2)  I just wanted to see what it can offer and how can I learn from it, or if it will be beneficial for me (48 physiotherapist age 40-60, <5 sessions, not applied, wave 2)  Basically what it was, a guided mindfulness session. I didn’t know how big it would be. Maybe I thought it was going to be more participants and I didn’t know if we would be able to see the practitioner or not, if she or he or the leader would be able to see us or if we would be able to see the other participants. I didn’t have a lot of clear ideas about what it was going to be like… I have done one at work before. I wasn’t quite sure what to expect (14 manager age 40-60, <5 sessions, not applied, wave 1) |
| Anticipated unspecified positive benefits | I was expecting it to be helpful and easily accessible and easy to do (04 nurse practitioner age <40, 5-10 sessions, applied, wave 1) | I didn’t honestly think about it that much other than I know, at least on some level, that mindfulness practice would be good for me. That it’s something that I feel healthier when I’m engaging in it regularly. I just had a general positive expectation that just by showing up it would be a positive thing (10 physiotherapist age <40, 5-10 sessions, applied, wave 1) | see if it can actually translate into your everyday life. If you can actually understand more. Just have a better opinion of yourself as opposed to just related to work. To see if it can actually be something more beneficial outside of just work essentially (44 physician age 40-60, <5 sessions, not applied, wave 2)  I think it was a good way to try and see how this can benefit me in any way (44 physician age 40-60, <5 sessions, not applied, wave 2)  Feeling quite unmotivated, just finding things at work difficult for the winter and the early part of spring, so it wasn’t necessarily fatigue, it was more feeling a bit overwhelmed with work. The type of work I do has changed because of doing more things virtually. It’s more the fact that I’m working in a much more isolated way that is making it more difficult (75 physician age 40-60, # sessions, applied, wave 3)  I was hoping that it would help me sleep, it would help me be able to focus on the work I can, give me some tools for trying to get myself back in the right place of mind (78 physician age 40-60 #sessions applied wave 3) |  |

**Satisfaction and Importance**

| Theme | Age <40 | | Age 40-60 | |
| --- | --- | --- | --- | --- |
|  | Clinicians (physicians, nurse practitioners) | Allied health and other (physiotherapist, social worker, psychotherapist, admin asst, manager) | Clinicians (physicians, nurse practitioners) | Allied health and other (physiotherapist, social worker, psychotherapist, admin asst, manager) |
| Sessions met or surpassed expectations, found to be very beneficial | I guess it met my expectations because I wasn’t totally sure what it would look like and each time it’s different (01 physician age <40, 10+ sessions, applied, wave 1) | [in terms of relaxation benefit]  It was more than what I expected. I thought I would try, to help out with the burnout and with the pandemic. But it was a lot more relaxing than I thought it would be. I was able to get into a really deeply relaxed state. I don’t log on every week, so the weeks that I do, progressively the more I join, the easier it is to really get into that deeply relaxed state (22 psychotherapist age <40, <5 sessions, applied, wave 2)  [in terms of being welcomed and feeling less isolated]  Actually, it was way better than I expected. The facilitator, she was so great, she actually welcomed me by name…it was really warm. I was surprised she called me out like that, and I also thought that was really sweet and really thoughtful. It was just really good actually. It was really good (46 psychotherapist age <40, <5 sessions, applied, wave 2)  It feels really sort of caring, like a virtual hug (22 psychotherapist age <40, <5 sessions, applied, wave 2)  It’s just generally overall the focus of the noticing and sensations in the body. Just appreciating any discomfort and not fighting that so much. It’s more this really gentle attitude…it’s the overall spirit of it that I really like. And that’s hard to describe and I’m doing my best, but it’s the gesture, the fact that this initiative exists is an acknowledgment. And participating in it and everything else is just a bonus. It’s more like the spirit of it, beyond the specific tools, specific approach, specific exercise (46 psychotherapist age <40, <5 sessions, applied, wave 2) | I’ve had about two sessions. I thought they were actually pretty good. I think she used the expression ‘hurry up just to slow down’ or something like that. And actually, that really made sense. It’s like you always have to carve in time to take time for yourself and that was a really good way of putting it (44 physician age 40-60, <5 sessions, not applied, wave 2)  I can’t say there’s one part that really stood out more than the other. I think the whole thing from beginning to end was good (44 physician age 40-60, <5 sessions, not applied, wave 2)  Well it’s more than I expected because I didn’t expect it to be going on that long and I am grateful that it did because it took that time to learn some different mindfulness meditations (35 physician age 40-60, #sessions, applied, wave 3)  [score 10]  I needed multiple things to help me through this time and so it was very important to me to have that regular time twice a week, where I would check-in and do these techniques to make sense of what’s going on, being able to deal with the difficult situation. In order to keep working and go on in my life, it’s been one of the very important things for me (35 physician age 40-60, #sessions, applied, wave 3)  [score 8]  I found it very helpful, and it’s helped me manage some anxiety and worry. the availability on a continuous basis has been really helpful and I have benefited from it (75 physician age 40-60, # sessions, applied, wave 3)  [score 8]  I think it was pretty good. I think it was probably what I expected. I was a bit surprised by the fact that there were different teachers and they would all be somewhat different, but all emotionally equally helpful (78 physician age 40-60 #sessions applied wave 3) | Definitely a 10. It helps me reduce my anxiety. I think it is making me more of a well-rounded person. I am trying to focus on me, focus on my surroundings, focusing on here and now, not worrying so much about the future and just kind of bringing myself back to the here and now (02 admin asst age 40-60, <5 sessions, applied, wave 1)  I attend almost every session every week… I would say an 8. It gives me that focus. You know Monday to Thursday, during the work week, it gives me that opportunity just to come to a quiet space and be guided through an exercise to maintain calm and mindfulness. It’s very helpful (06 social worker age 40-60, 10+ sessions, applied, wave 1)  [score 8 for breathing exercise specifically]  It took me away from my extremely busy life, taught me to be selfish and concentrate on myself and able to focus on myself (48 physiotherapist age 40-60, <5 sessions, not applied, wave 2) |
| Relied on other supports in addition to P4P (family, other meditation or mindfulness) | [score of 7] It’s very nice to have and it’s been particularly useful because of the timing of how it came up in the midst of a pandemic. If it didn’t exist, I have other practices, and there is other ways to continue in this type of practice or with this type of community. So I would say not the most important thing but it’s been a nice consistent part of my routine (01 physician age <40, 10+ sessions, applied, wave 1) | [score 6] I have attended semi regularly but it’s been regular enough that it’s had quite a positive impact. And yet there is probably even room to benefit more with more consistent attendance and practice. I think I have relatively good balance in my work life and outside of work life as well. I find that I have other skills, outlets and support. I think this is one of several very positive supports that I am fortunate to have (10 physiotherapist age <40, 5-10 sessions, applied, wave 1)  [score 9] I’m not giving it a 10 because there are other aspects in my life contributing to positive mental health. But this program is really important in terms of the mindfulness that I’m able to implement during the week and remind myself of it, the improved sleep. And just overall allowing myself to go into that calming safe space and quiet my mind. It’s just really important to having balance for me (22 psychotherapist age <40, <5 sessions, applied, wave 2)  [score 7]  I think that type of mindfulness session is helpful for personal wellness and stress reduction on the job and productivity and all of those things, if it’s done in conjunction with other self-care. I also go to counseling, for example, and all of these other things that I do for myself. So I think it’s very important, but on its own, I don’t think it meets the need to take care of ourselves as helpers (76 admin assist, age <40, # sessions, applied, wave 3) |  |  |
| Feels less needed than at beginning of pandemic | [score of 6] anything less than 5 would portray that it was unhelpful. But why 6 and not like an 8? I found over time…maybe I’m just managing better and I just don’t need it. I would say probably if you asked me how important it was to me in the month of April, I would have given it an 8 and how important it is to me now I feel like it’s a 6 (04 nurse practitioner age <40, 5-10 sessions, applied, wave 1) |  |  |  |
| Having done few sessions, too early to assess value |  |  | I can’t say yet. I think I’m still pretty new. I’ve only done two sessions. Yes and no. In the sense that I think it’s nice to have that put in to your schedule a little bit but I can’t say that it’s translated into anything else in my life yet (44 physician age 40-60, <5 sessions, not applied, wave 2)  I say 5 is because it’s something that I’m curious about. I hear about this. I think I need to learn more about it. So this is a great opportunity. So it’s not 0. But at the same time, it’s not 10 because I can’t say that I’m a star and know exactly what it is, and I’m versed in it. I’m kind of still trying to figure this out (44 physician age 40-60, <5 sessions, not applied, wave 2)  I’ve barely scratched the surface…it’s the beginning of a learning. I’m hoping that if and when it ends, I will be able to continue this. A 10 out of 10 would be a sort of life-changing, I don’t think it’s life-changing. I would call it life-enhancing (78 physician age 40-60 #sessions applied wave 3) | I don’t realize if it’s helped me as I said I only did one session (48 physiotherapist age 40-60, <5 sessions, not applied, wave 2)  It’s been only less than a week since I took my first session so I just haven’t had time yet but I think I also need to do more than one. Once I’ve got a good session under my belt, then I’ll have more tools to work from (14 manager age 40-60, <5 sessions, not applied, wave 1)  I have only done it once so a 6 or a 7. I definitely want to do it again. I don’t think my life has changed drastically but I think it could certainly help me if I stuck with it (14 manager age 40-60, <5 sessions, not applied, wave 1) |

**Impact**

| Theme | Age <40 | | Age 40-60 | |
| --- | --- | --- | --- | --- |
|  | Clinicians (physicians, nurse practitioners) | Allied health and other (physiotherapist, social worker, psychotherapist, admin asst, manager) | Clinicians (physicians, nurse practitioners) | Allied health and other (physiotherapist, social worker, psychotherapist, admin asst, manager) |
| Detach from work |  | The fact that there is a start and end time on two specific dates that’s helpful because not only do I need to be there for the start time but it’s also time away from other activities that I’m up to. Just that gesture of stepping back or stepping aside, I found that really useful (46 psychotherapist age <40, <5 sessions, applied, wave 2)  With my work I find because there is much less structure to the day, start time to the day, any breaks or lunch, and end time to the day, there really is nothing like that unless I structure that on my own and really have a lot of discipline and stick to it. There are no real anchors and I thought this might be one so I could work towards structure with the final 8.30 pm event in mind. There is something to work towards and something to orient me (46 psychotherapist age <40, <5 sessions, applied, wave 2)  I had this hyper-vigilance or external response that’s really definitely gotten worse and definitely sort of a plateau where I’m still having a hard time managing that… Even just taking that step to participate is really helpful for me. I’m hoping over time that the side effects of the actual exercises will also just have an effect (46 psychotherapist age <40, <5 sessions, applied, wave 2) | it helps me to realize it’s not all about just going, going, going and bringing home your work and thinking about it 24/7. I think it’s a good way to almost detach a little bit from that stuff. And that helps you to not think of your work as often as you do (44 physician age 40-60, <5 sessions, not applied, wave 2)  it’s busy so between operations, which need to be done within the next two weeks, between bringing home some charts because you have surgery the next day…we do bring home work a lot, so I think what I liked about is that…It was the half an hour to not do that. To actually slow down your mind, slow down everything. I realize how hard it is to do that. It was nice to be able to do that by following her instructions. You don’t realize how much your mind keeps going until you do a session like that (44 physician age 40-60, <5 sessions, not applied, wave 2)  I don’t think it’s the stress. It’s more the preoccupation of things. I think it’s more because your mind is constantly going, your mind is constantly doing things, and we have a lot more to think about in the last year. I think it’s helped to just give yourself a little bit more of a slowdown. I can’t [inaudible] otherwise I would be doing this every day. For me it’s more learning about things as opposed to being [15:30] oh my gosh I have so much stress and I don’t know how to deal with it (44 physician age 40-60, <5 sessions, not applied, wave 2)  It’s half an hour when you can just focus on yourself, but in a way that’s not work-related, it’s just about you looking inside, getting to know yourself and focus your mind in a place (78 physician age 40-60 #sessions applied wave 3) | in the early stages of the pandemic there was just a lot of uncertainty kind of everyday, as everything unfolded, and we were starting to understand it better and it was just a lot of information. I think having that time to be grounded has allowed me filter out what information I needed to pay attention for myself as opposed to all the other information that was out there. It just kept things contained so that my anxiety was very manageable (06 social worker age 40-60, 10+ sessions, applied, wave 1) |
| Time to for self at end of day to unwind and relax, sleep better | Overall wellbeing. I don’t know if that’s the right word, but it’s been a stressful, emotional time for many of us. And so, for me personally, having a time in your day that you are carving out to essentially do nothing, to just sit there and observe. it’s causing you to pause and have more of an inward reflection, notice what’s there instead of having that pressure to always be running around and doing a lot of things. it’s beneficial for both clinical practice and preventing burnout. And then also in your overall health or in your overall balance in your life (01 physician age <40, 10+ sessions, applied, wave 1)  Maybe every few days. Certainly, around end of day, winding down, trying to get settled for sleep. I do often recall some of the practices that we did like the breathing space one (04 nurse practitioner age <40, 5-10 sessions, applied, wave 1)  it can help with stress management, it can help with things like sleep because especially if you are doing in the evening, after you have eaten dinner and you are winding down for the night, it can often help you have a better quality of sleep (01 physician age <40, 10+ sessions, applied, wave 1) | It’s definitely easier to get into that relaxed state to go to sleep…I definitely feel more relaxed those nights when we have the session (22 psychotherapist age <40, <5 sessions, applied, wave 2) | It helps with my sleep too (35 physician age 40-60, #sessions, applied, wave 3)  It probably has helped a bit with sleep onset as well so I think I fall asleep a bit easier. If I’m feeling anxious before I sleep to try and concentrate on my breath and say okay I can put whatever is going on inside my head away and just focus on the breath and try to transition to sleep (75 physician age 40-60, # sessions, applied, wave 3)  I sleep better. Allowing yourself to not carry everything home lets you sleep better. I just use them if I’m going to bed in the evening and I’m feeling caught up in something, I’ll use that just to sort of clear my mind which helps you sleep (78 physician age 40-60 #sessions applied wave 3) | Well certainly after the half hour I was just more relaxed and less rushed. I think probably I was able to sleep a bit better because of that. I didn’t go to bed overthinking things (06 social worker age 40-60, 10+ sessions, applied, wave 1)  I think it helped me as a person. As a person who is stressed out. The importance to realize that sometimes I need half an hour on my own. Not just taking care of everybody else but be a little bit selfish and concentrating on myself (48 physiotherapist age 40-60, <5 sessions, not applied, wave 2) |
| More present/calm at work or in daily life | Patients right now are very stressed out with everything going on, there is a lot of frustration. They are upset sometimes that they can’t be seen in person, they’re upset that certain investigations and tests are delayed. So, at least for me, I find we are dealing with people who very frustrated and emotional. And so that is even more reason that you have to be that steady, grounding presence and not get super reactive in this situation. Having a consistent mindfulness practice is extremely useful in being able to stay grounded even when everybody else is very, very distressed (01 physician age <40, 10+ sessions, applied, wave 1) | Right now in our healthcare environment, there is constantly being new things asked of us that are outside of our usual scope. My automatic reaction might more of annoyance or resistance. Noticing what that feels like in the moment, I have been able to respond a bit more thoughtfully, considering and weighing a little bit of my priorities in the moment or what someone’s else perspective might be, what the needs of others might be. It’s helped me to respond a bit more thoughtfully in moments of being sort of asked to do something that maybe is outside of my ability or my scope or my comfort at the time (10 physiotherapist age <40, 5-10 sessions, applied, wave 1)  When we are practicing mindfulness, we are more present when we are helping people, we can listen more, we can hear what they are actually saying, we have more patience to understand what they are going through and come up with a plan of care (76 admin assist, age <40, # sessions, applied, wave 3) | It helped me be more calm if there is a very stressful situation at work, be able to think better and work better with other people, be more aware of what is going on and work better with a team…it helps with resilience (35 physician age 40-60, #sessions, applied, wave 3)  I think I’m better with my daughter, getting along with her, understanding and also having more compassion to myself and others (35 physician age 40-60, #sessions, applied, wave 3)  [The sessions] help me to calm down a bit, I just recognize what’s going on in my brain and how to respond. I am trying to respond to situations rather than react, which I’ve done a lot in the past, and I still do, but I’m trying to learn how to respond when adversity comes along or people say things I don’t like, some mindfulness can help the way I think (35 physician age 40-60, #sessions, applied, wave 3)  It has helped me decrease my anxiety, it has helped me be more mindful during the day, I am better able to slow my brain down and quiet things down by just pausing and taking a couple of breath. And when there is an opportunity to relax, I am better able to take advantage of it now, so I can actually pause myself and say, okay, you can take three breaths now and try to slow down (75 physician age 40-60, # sessions, applied, wave 3)  I think there’s less stress, but I’m not quite sure if it’s just because we’re so good at ramping up and down from surgery now than during the first troubled times, but I think it’s helped with my stress (78 physician age 40-60 #sessions applied wave 3)  It’s allowed me to be a bit kinder, I guess, which is kind of an odd thing to say. Less confrontational, not that I was particularly confrontational, just the basic premise of my personality, but you know accepting of other people’s points of view. When someone would have annoyed me, it doesn’t annoy me as much (78 physician age 40-60 #sessions applied wave 3)  I’m much more productive with my time…and when you start to get more things done you don’t stress as much about it. It’s still pretty intense and it’s been a year and half…it just made me more level and able to work (78 physician age 40-60 #sessions applied wave 3) | I tend to be a bit more relaxed now I have found. I’m not so uptight. It just kind of gives me a better understanding of myself and to focus more on the here and now, and not worry about the future and if I start getting a bit anxious just refocus, redirect myself with the suggestions (02 admin asst age 40-60, <5 sessions, applied, wave 1)  After doing the mindfulness I just don’t have that urgency or that tendency to overthink situations. I think in that regard I am much calmer (06 social worker age 40-60, 10+ sessions, applied, wave 1)  Pausing felt good. I did feel more centered and calmer after. Actually, whether or not the session itself helped, it was just knowing that I feel kind of lighter that I have this resource (14 manager age 40-60, <5 sessions, not applied, wave 1)  It helps me kind of focus in the here and now and present. It also helps me that… What I found is that a couple of them they gave tips and advice about, if you feel like your thoughts are kind of getting ahead of you try putting yourself back into the present, trying to refocus with your breathing and everything. So that has kind of helped me at work when things have gotten a bit stressful (02 admin asst age 40-60, <5 sessions, applied, wave 1)  I have become a bit calmer. I can deal with things a bit better than at the start of COVID. At the start of COVID everything was just coming at me and I got to the point that I couldn’t deal with things anymore. I had been doing the mindful sessions and with the tips if you feel like you start to get overwhelmed, take a step back, breathe, or do the 5 senses like the touching, seeing, etcetera, smelling and it’s kind of coming back to the here and now so I can deal better. I can deal with the patients better. I’ve got a little bit more of an understanding for the patients and I’m not so, let’s say judgmental towards them. I can handle them better (02 admin asst age 40-60, <5 sessions, applied, wave 1)  Dealing with patients that tend to be very anxious and 300 questions as to when will this all end and you know trying to… And then trying to understand COVID and posing 301 questions to you can be challenging and the patient’s anxiety level and them refusing to wear a mask etcetera that kind of gets a little bit stressful, it keeps happening day in and day out and then it’s like time for me to step back and use the practice to just calm myself down (02 admin asst age 40-60, <5 sessions, applied, wave 1)  It has allowed me to really focus on my grounding and be more focused in the moment. I think by doing that it’s allowed me to better maintain my interactions with other people because I’m not caught up in the anxieties of the state of the world or healthcare system concerns with the pandemic (06 social worker age 40-60, 10+ sessions, applied, wave 1)  It opened up my judgement with it and it gave me a bit of a different perspective that a lot of it is mind over matter and if you just focus and your breath and you concentrate on the here and now it helps you get through things better (02 admin asst age 40-60, <5 sessions, applied, wave 1) |
| Felt equipped with toolbox to invoke when needed | I just feel better armed to deal with… I think I have more tools in my own toolbox to pass on to others, I guess. Just feeling like a confidence in incorporating some of this into my own practice (04 nurse practitioner age <40, 5-10 sessions, applied, wave 1) | I understand that mindfulness practice with experience you can better notice your own thoughts and reactions and be able to respond more thoughtfully and more mindfully. I think any reminder of that sort of skill at some point translates over into my daily life. It’s constant. It has to be something I keep on top of mind to use the skills. But having a regular forum for practicing, and having some options, I think just makes it easier to access those skills in moments where a mindful response is really needed or really useful (10 physiotherapist age <40, 5-10 sessions, applied, wave 1)  it’s just general practice of mindfulness skills, outside of specific need to practice it’s just a healthy habit in maintaining general awareness and presence in day-to-day moment… overall, it’s just a healthy thing to be practicing and it’s good reminders for using day-to-day skills, practice (10 physiotherapist age <40, 5-10 sessions, applied, wave 1)  Starting to put some tools in place. Really practical ones to try and mitigate some of that stuff. Just in terms of the nervous system activation…with all this chaos it’s really easy to become distracted and not have any of my own practices. This is really good in bringing back to that the importance of how to actually use them (46 psychotherapist age <40, <5 sessions, applied, wave 2)  It’s helped me with my everyday stressful moments, just having little tools that as the facilitator talks about you know, taking really deep breaths throughout the day when something stressful happens so it helps improve my performance at work and reduce my stress (76 admin assist, age <40, # sessions, applied, wave 3) | [The sessions] allow me with the courage to go into something even when my brain is telling me all sorts of bad stuff and I can still just keep going on helped (35 physician age 40-60, #sessions, applied, wave 3)  I think it’s given me the ability to accept the things I can’t change, the external things I don’t have control over. I’m trying to be a bit more accepting of that. I guess I’m a little bit type A and controlling of my environment. And so things that are outside of my environment would bother me, and I’ve sort of learned to be much more accepting of that. It’s helped me when I’m hitting a road block, and I’m able to stop and take 5 or 10 minutes and do the little S, T, O, P drill, and give myself a few moments to gather myself and then move forward again (78 physician age 40-60 #sessions applied wave 3) | It’s good to know that is a resource out there and that I find it valuable so that I can go back and do it again. I have a solid job but it’s still very, very stressful and I’m not good at taking time to think about it and to actively address it, just let the stresses pile up (14 manager age 40-60, <5 sessions, not applied, wave 1)  They give you strategies to be able to use the tools in a very quick manner, so if you’re feeling stressed at work, you just do a breathing strategy. So you are just trying to quickly use a tool to get yourself more grounded and calm if you’re feeling very stressed in a situation (79 other age 40-60 applied wave 3) |
| Actively applied mindfulness to better manage stress at work | When I tend to find that I’m being hard on myself, I do hear Dr. <name’s> voice talking about the loving kindness because I think that is one of the themes that is paramount to her sessions. I feel if I am having a particularly low day or moment I sometimes, and I have actually started deliberately practicing. Someone at one point talked about showing yourself grace and compassion and I think that is something. I probably think about that every day, either as it applies to myself like showing myself grace and compassion or someone else. Someone that maybe is making my life harder than it should be (04 nurse practitioner age <40, 5-10 sessions, applied, wave 1)  I usually do it once a week and it’s been a nice grounding practice to try and stay consistent with that even with everything else changing so much in the last few months (01 physician age <40, 10+ sessions, applied, wave 1)  The micro practices, they’re nice and short, so they’re just something you can use when you’re not having a formal meditation. I could probably use them more. I don’t know maybe once a week, it depends how work is going. If I’m more stressed, I will think to use it more because you are thinking, oh I’m feeling really rushed or frazzled, okay I should do something. Whereas, if it’s a time at work where things are relatively okay, then you don’t always think, oh maybe I can throw in one of those micro practices. So, I would say kind of varies (01 physician age <40, 10+ sessions, applied, wave 1)  Being present, being consistent and calm when you are working in very high stress environments. Sometimes in the sessions they might give you some ideas, I think Dr. <name> calls it micro practice. Basically, something that you can do that takes less than a minute. For instance, she had one that was name 5 things that you can see, 4 things you can touch, 3 things you can smell, 2 things you can hear, one thing you can take, something like that. Basically, a little exercise. And Dr. <name> has some different mnemonics about ways that you can pause, not just when you are having a proper meditation, but actually in the middle of your day, if you can just do this thing for a minute. And I did try that out when I was at work, and especially when you’re feeling overwhelmed or just tired. And so, I think just being able to use those skills can help you think more clearly, tackle things in a calmer manner (01 physician age <40, 10+ sessions, applied, wave 1)  Patients right now are very stressed out with everything going on, there is a lot of frustration. They are upset sometimes that they can’t be seen in person, they’re upset that certain investigations and tests are delayed. So, at least for me, I find we are dealing with people who very frustrated and emotional. And so that is even more reason that you have to be that steady, grounding presence and not get super reactive in this situation. Having a consistent mindfulness practice is extremely useful in being able to stay grounded even when everybody else is very, very distressed (01 physician age <40, 10+ sessions, applied, wave 1) | Yes, I have…about 2 to 3 times a week. I think never as consistently as I’d like but really the answer is yes, I have continued practicing…I can’t think of specific practices but more so the spirit of just stopping and paying attention to what my thoughts or particular worries might be in a moment. And I say again, with a self-compassionate lens I think trying to change the automatic response from either a self-blaming or a striving mind state. I think maybe it’s more of a self-compassion reframing that I might use as a skill. That has been more of a theme than a specific practice (10 physiotherapist age <40, 5-10 sessions, applied, wave 1)  I also noticed that I have a little bit more of a key sense of awareness to use some of the mindfulness techniques throughout my day, that are practiced during the Pause 4 Providers sessions. Now, I’m not saying I’m doing it all day everyday, but I definitely notice myself from time to time reminding myself to be aware of my feet on the floor and my back pressing up against the chair, and shades in a painting that I might be walking by or walking across… Just being more aware of my breath and my breathing, my lungs filling up… And time and appreciation for my body that these organs are working for me (22 psychotherapist age <40, <5 sessions, applied, wave 2)  Even throughout the day I think of some of the exercises. I’m seeing that start to change in a positive way. I’m taking a break from work at my desk, I stand up and I’ll take a moment with the practices in mind (46 psychotherapist age <40, <5 sessions, applied, wave 2)  Grounding techniques. The facilitator mentioned tuning into your five senses, what are five things you can see, what are five things you can smell, five things you can taste, and doing that throughout the day has been helpful along with just breathing. The five senses thing, I’ll do that and do breathing throughout the day when I’m getting stressed at work or if I’m really frustrated or I’m anxious. And the self-compassion, you use just throughout the day as well or when you’re meditating (76 admin assist, age <40, # sessions, applied, wave 3) | The small little things like the strong back, soft front, that I can do quickly before something super stressful, or the stop one or the rain…just taking one breath at a time to recognize what is going on, that has helped (35 physician age 40-60, #sessions, applied, wave 3)  Definitely the idea of three breaths and attending to just paying attention of the breath itself as a way of calming myself down is something that I do much more frequently now than before (75 physician age 40-60, # sessions, applied, wave 3)  I like the body scan. And there was one session where they talk about how, when you get upset, your mind always goes back to the big troubles you are having, and how you can focus to shut those down. And giving yourself permission to know that you don’t get everything right all the time and you do your best, those kinds of things are helpful (78 physician age 40-60 #sessions applied wave 3) | During the course at work, probably 3 or 4 times during the course of the day. Not really much so on the weekends. Most likely Monday to Friday at least 3 to 4 times a day (02 admin asst age 40-60, <5 sessions, applied, wave 1)  It has allowed me to further develop my mindfulness practice using different techniques that they [inaudible] within their practice. It’s a learning opportunity and I just really appreciate that (06 social worker age 40-60, 10+ sessions, applied, wave 1)  On Tuesday nights, <name> often talks about a micro practice to do the next day. Take those ideas that she talks about and incorporate those into my daily activities… I use those like on the non-Pause 4 Providers days, on the Friday, Saturday, Sunday, or just generally during the day (06 social worker age 40-60, 10+ sessions, applied, wave 1)  I kind of take reflections that we have talked about from the program and reflect on them during the day. I am attending four days a week but probably up to 6 days a week. Maybe more. Sometimes it just becomes part of your regular daily routine you don’t think that you’re incorporating those practices (06 social worker age 40-60, 10+ sessions, applied, wave 1)  Almost every day, one of the small practices, because I have a lot of trouble with sleeping right now, I think from stress and stuff, so I’ll use those strategies in the middle of the night sometimes, and at work if I’m feeling really stressed out (79 other age 40-60 applied wave 3) |
| Shared mindfulness techniques with others (e.g. co-workers, patients, family) | I actually lined up a bunch of mindfulness tips that I have been able to use with patients. Which was a surprising outcome perhaps even an unintended or unanticipated outcome. Llike a quick opening breathing exercise, those have been really helpful to use with my own patients and even with my kids (04 nurse practitioner age <40, 5-10 sessions, applied, wave 1) | The same thing goes for being mindful and thoughtful when speaking to family members, people that I’m close to. It can happen naturally any time where we may have a reflex to react a certain way or to be irritable in moments of stress. I think it does help and has helped me decrease the automatic emotional reactivity at times where maybe otherwise I wouldn’t of taken that extra moment to think (10 physiotherapist age <40, 5-10 sessions, applied, wave 1)  I work in an area where mindfulness practice is often recommended for patients as well. It’s part of our daily offerings of services. It’s something I have awareness of. But I think just some increase in awareness of the benefits and getting to experience them personally again, or in a new way, helps me to speak that language with patients that might be going through that type of program. Or if I think they could benefit from a mindfulness program, it is helpful and more authentic to have some personal experience that’s more recent. Just to relate that to patients through my own experience, how I feel it’s benefited me (10 physiotherapist age <40, 5-10 sessions, applied, wave 1) |  | It was good as a leader to let staff know that I thought this was important and to be able to say that I had done it myself so that I could encourage them to do it and say that it was worthwhile…I talked about it with a few other people that I work with. It was nice to be open to having those conversations with other people (14 manager age 40-60, <5 sessions, not applied, wave 1) |
| Reduced feelings of isolation and loneliness | Particularly in March. I started pretty close to the first week I think, or maybe the second week. Especially at that time, it felt almost like a symbolic gesture, like all these doctors coming together after a busy day and a very stressful time to sit together. And specially in the earlier sessions, I thought that was quite powerful in terms of feeling connected. Feeling connected also to physicians that may be in different specialties and working in different cities because sometimes we get a little bit stuck in the bubble of our hospital or the bubble of our specialty. When I went to the first session, I didn’t know any of the other people but you still kind of felt, even if you don’t know them everyone is facing similar challenges and probably having similar worries. That was nice to sit together. Also, in the beginning or at least the first two times I did it, people did put their cameras on, so I did put my camera on and then you could see the other faces. But then, certainly the overwhelming trend after those first two is everyone just has their cameras off the whole time. And then maybe once in a while if they ask a question, they might unmute themselves or they might turn their camera on. I think when everyone has their camera on it’s sort of nice but then if nobody does it then you feel weird being the only one with your camera on]. I noticed that kind of effects that sense of connectedness (01 physician age <40, 10+ sessions, applied, wave 1) |  | It’s really comforting to see the same people coming, even if I don’t see them, sometimes I see their comments…I always like the idea of a community so feeling included or part of the group, where I’m not lost in a big system, so that helps (35 physician age 40-60, #sessions, applied, wave 3) |  |
| Grateful that P4P acknowledged the needs of healthcare workers |  | It’s really nice to have that acknowledgement, that this external party is recognizing that healthcare workers are human beings that have needs as well. We’re not robots that can just keep on going. We feel the impacts of the global stressors at large, let alone a pandemic, on any given day. We’re part of society too, we’re people too that are having a hard time with this. It was really nice to have this external party say I see you, we know, we see it, we know that this is hard, and we know that you’re probably burnt out and we’re here at no cost to you and reliably week after week, twice a week. It almost felt like a service to help you out (22 psychotherapist age <40, <5 sessions, applied, wave 2) |  |  |

**Design**

| Theme | Age <40 | | Age 40-60 | |
| --- | --- | --- | --- | --- |
|  | Clinicians (physicians, nurse practitioners) | Allied health and other (physiotherapist, social worker, psychotherapist, admin asst, manager) | Clinicians (physicians, nurse practitioners) | Allied health and other (physiotherapist, social worker, psychotherapist, admin asst, manager) |
| Participation is easy (virtual, easily accessible, drop-in so no advance registration or screening) | I love that it’s drop-in, you don’t have to register or sign up or make a plan (04 nurse practitioner age <40, 5-10 sessions, applied, wave 1) | Yes, it is what I expected. It has been reliable and consistent in that it’s the same process each week. When I can make it, I just go to the website and sign in. That’s very comforting and easy in that there is no organization required to participate, so I think that even exceeds expectations (10 physiotherapist age <40, 5-10 sessions, applied, wave 1) | there was a no judgement thing so I didn’t have any reservations at all… nobody asked questions (44 physician age 40-60, <5 sessions, not applied, wave 2)  It’s easy to participate, you can just do it from home. I think the ability to attend some sessions and not others is nice too. I feel like if we make diner late and I’m still cleaning up at 8.45, I don’t really feel bad that I haven’t attended that session, but I do really try to make an effort on the days the sessions are on to get things done a bit earlier (75 physician age 40-60, # sessions, applied, wave 3) | I could log on if I wanted or not log on if I didn’t want, or discontinue midway, though there is no concerns. I don’t keep my camera on, nor obviously my volume (06 social worker age 40-60, 10+ sessions, applied, wave 1) |
| Variety of activities in each session or across sessions | Depending on which evening you went to, you kind of got a different flavor of mindfulness. I found it actually quite helpful that you could kind of try out different evenings and find a style or a therapist that works for you and then continue coming that same night (04 nurse practitioner age <40, 5-10 sessions, applied, wave 1)  I did also enjoy little interludes as well, a poem or a thoughtful phrase here and there. I joined a couple sessions where it seemed to flow really nicely. I thought that there was kind of an initial activity and then a little interlude moment and then a second activity (04 nurse practitioner age <40, 5-10 sessions, applied, wave 1)  If you aren’t meditating regularly, just sitting still for 30 minutes with long stretches of silence is pretty difficult, but I found the 30 minutes was kind of broken up. Dr. <name> would often do an introduction and then she would do a longer practice, maybe 15 or 20 minutes, and then kind of pause, and people could ask questions, and then she’d kind of end with a closing practice. Even in the longer practice, it wouldn’t just be one thing, like it might start with some components of movement or stretching, and then move on to a different practice like a body scan or a breath meditation. It was broken down into smaller chunks so I thought that makes it more accessible for people including myself (01 physician age <40, 10+ sessions, applied, wave 1) | Each week’s been a little of a different focus, so I think there is always new practices or new ideas that you don’t necessarily expect but are always positive and interesting. I’d say on the whole, it has been as advertised in that it has provided even broader practice options and items to reflect on than expected (10 physiotherapist age <40, 5-10 sessions, applied, wave 1)  There is often more than one practice. For example, there could be a movement practice and more of a body focused practice and a little reflection on that. And then something that’s more sort of looking inward, noticing thoughts, noticing thinking patterns. There are different focuses in the practices that they choose. Some are making you work more towards self-compassion specifically or compassion towards others. I think there’s just a broad spectrum of different things to be mindful of (10 physiotherapist age <40, 5-10 sessions, applied, wave 1)  One of the other nights there was a poem shared near the end. That was a nice transition coming out of the meditative state, opening our eyes and transitioning with the poem from the meditative state to opening the floor to any questions. The poem was a nice transition (22 psychotherapist age <40, <5 sessions, applied, wave 2) | Learn different things like the mountain one, the body scan, there was a lake one recently, the one that she does with the art, and the compassion…yes, I like the variety and also the repetition (35 physician age 40-60, #sessions, applied, wave 3)  They all have a different way of doing the mindfulness, so it’s good variety (35 physician age 40-60, #sessions, applied, wave 3)  Some are more focused on physical things like relaxing muscles and those kinds of things. And there are other things that are more about giving yourself permission to make mistakes and forgive yourself, and not be so focused on being right or perfect all the time (78 physician age 40-60 #sessions applied wave 3) | In each of the four sessions, there is always something different that they work on. One will focus on a picture, another one will focus more on working through your body. Each technique is good and it’s nice to experiment with different types of techniques so if one doesn’t work you can always think of trying another technique. If working on the sense, you know 5 things that you see, 3 things that you touch or whatever, if that doesn’t work you can try focusing on your breathing, if that doesn’t work you can try refocusing your energy and stare at a picture and study the picture and just try to get your mind at ease (02 admin asst age 40-60, <5 sessions, applied, wave 1)  I like the variety. Monday nights is more of a mindfulness through visualization. And the instructors they each do something different, and I enjoy the differences of each facilitator’s approach to mindfulness (06 social worker age 40-60, 10+ sessions, applied, wave 1)  The facilitators have different approaches. I just appreciate that there are many ways to do things and as a result there are also many ways to present mindfulness. I just appreciate the differences that each of the facilitators brings to it so I can’t say there is a favorite part (06 social worker age 40-60, 10+ sessions, applied, wave 1) |
| Some interactivity, shared experience |  | I actually really like the way it begins. I’m thinking in particular of everyone landing in the session and coming from, I’m just imagining, all the places they are at. It’s almost instantly switching into a significantly more relaxed state. That really stands out because of the change from chaos in the day to, okay I’ve set aside a time, I’ve set that space and now welcomed in sharing this experience with peers. That really stands out (46 psychotherapist age <40, <5 sessions, applied, wave 2) |  | Sometimes people will either make comments verbally or within the chat. It’s just interesting to see what other healthcare providers’ reflections are on the topic we’re all involved with (06 social worker age 40-60, 10+ sessions, applied, wave 1)  I really also enjoyed that there was a little bit of dialogue at the end, and I thought that was so nice for other people to share their comments, to be able to share mine (14 manager age 40-60, <5 sessions, not applied, wave 1)  It was actually nice to be able to thank the instructor. I did find it valuable and I wanted people to know. I think it’s important to show our appreciation right now, so I enjoyed that piece of it (14 manager age 40-60, <5 sessions, not applied, wave 1)  Generally, with self-care it’s the self aspect, right? And that we’re in it on our own but I liked seeing just the names of other people that were involved. It’s kind of nice. I knew that other people were in the same boat but it’s nice to see them and to see us all kind of coming together and taking care of ourselves. I found that comforting (14 manager age 40-60, <5 sessions, not applied, wave 1) |
| Specific concepts or techniques viewed as beneficial | [breathing, imagery]  In particular 5, 4, 3, 2, 1 or a breathing space. And I also did a few sessions with Dr. <name> where she used imagery. She put up a painting and had you focus and explore and be curious of different parts of the painting. I think of that often especially when I’m looking at a photograph or piece of art (04 nurse practitioner age <40, 5-10 sessions, applied, wave 1)  [Seeing the familiar in a new way]  They had us look around the room, which of course is a room in our house but look at it in a way that you are describing it to someone else and so, you are looking at things that are very familiar but kind of seeing it in a new way because there is a lot of time you aren’t really paying attention to what is around you (01 physician age <40, 10+ sessions, applied, wave 1)  [Concluding/Take-away reflection]  Sometimes they will end with a poem or a short passage, and they just read it out, and after sitting in silence, that can be a really nice way to tie things together. Depending on what the poem or reading was about, it gives you some food for thought that you can take after the session to kind of think about and reflect on. The quotes or the poems or the passages that were included, I really enjoyed that element of it (01 physician age <40, 10+ sessions, applied, wave 1)  [scientific mechanism of action]  I think some of the mindfulness explanations that were really ‘science-y’. I actually really appreciated talking about how our amygdala is firing up and reptilian brain and all of that. I found it really interesting, and it’s helped me to recognize it in myself when I need to come out of my head and into my body. I think just feeling really busy or really stressful or there’s too many demands on me, taking a moment to actually practice (04 nurse practitioner age <40, 5-10 sessions, applied, wave 1)  [scientific mechanism of action]  I did like the explanations of what physiologically is going on in our bodies when we’re feeling in that fight or flight place. I think that reminding us that we are also humans, and this is how the human body works, it was helpful (04 nurse practitioner age <40, 5-10 sessions, applied, wave 1) | [compassion, self and others]  My experience has been with mindfulness-based stress reduction and so I’ve had those specific practices in mind only. I think I’ve had less experience with self-compassion specifically. I think it’s probably a nice choice for where we’re at right now. I would include that as a positive new experience (10 physiotherapist age <40, 5-10 sessions, applied, wave 1)  [compassion, self and others]  Self-compassion practices are probably the ones that stick out most. Maybe they come up often and maybe they feel like they particularly resonate with me and the other participants. I think more of just that style of practice sticks out because they set pretty much the whole point or a big part of the purpose of this program which is finding some space for self-care amidst chaos (10 physiotherapist age <40, 5-10 sessions, applied, wave 1)  [compassion, self and others]  The whole practice was around being kind to yourself and kind to others even though we may differ in our opinions or views etcetera. Still wishing health and happiness for that person because there are deserving of it. That was just very nice, impactful. And she also talked about healing for the self as well. And that was really impactful given the social-political state the world is in right now, so much divide and social injustice and political divide. It was really refreshing to talk about community and generosity and focus on that, when we’re bombarded everyday with the news and tv about what’s different about us right now (22 psychotherapist age <40, <5 sessions, applied, wave 2)  [imagery]  She used a painting for the mindfulness practice which was really interesting. She said that she was trying something new. And I really liked that practice of not just using our mind and being quiet but actually keeping our eyes open and engaging with something that we might not have even given a second thought to, a painting. Or just taken a quick look at it and kind of decided nice or not nice. Maybe what speaks to you about it and move on. But she spent a good probably 20 minutes on this painting and appreciating the textures and colors and shadows, the postures of the people, where the painting was done, the culture (22 psychotherapist age <40, <5 sessions, applied, wave 2)  [body awareness]  I’m thinking of in particular are these reminders of returning to the knowledge I already have about like body scan, a lot of mindfulness exercises, this visualization or just really sensory kind of practices. Just redoing that and revisiting it, and then guiding us through it. Yes, that’s practical. She kind of gives you a new perspective and a new angle (46 psychotherapist age <40, <5 sessions, applied, wave 2)  [self-compassion]  I really appreciated an approach called mindful self-compassion because that is something that I am familiar with and I have practiced before. I was happy that approach was incorporated (76 admin assist, age <40, # sessions, applied, wave 3) | [breathing]  The most important thing was to try and get back to the breathing…the second thing is…she went through going from your head to your toe. You’re being mindful. I think it’s the way it’s putting your mind onto you and parts of you as opposed to getting the mind to wander (44 physician age 40-60, <5 sessions, not applied, wave 2)  [slowing down]  She said something like you’re hurrying up to slow down. Hurry up to slow down. And I thought that was an interesting expression… My feeling was that you have to take the time to find time for yourself (44 physician age 40-60, <5 sessions, not applied, wave 2)  [visualization, compassion]  I like the mountain one and I tried the lake one…and then the self-compassion break, when I’m struggling with something terrible I go to that, eventually it’s pretty helpful (35 physician age 40-60, #sessions, applied, wave 3)  [art]  One of the facilitators does art-based mindfulness sessions, that’s not something that I really expected and I found that kind of interesting and it was novel for me (75 physician age 40-60, # sessions, applied, wave 3)  I learned that I work much better from the audio methodologies than the visual. I had no idea. Because one of the [facilitators] focuses on art and meditating. It didn’t speak to me as well as doing it more cognitively and with voice (78 physician age 40-60 #sessions applied wave 3)  [body scan/self-compassion]  I like the body scan piece because it makes you think about parts of your body that you don’t normally think about. Self-compassion was actually another interesting exercise (78 physician age 40-60 #sessions applied wave 3) | [strategies for being present]  Yes, it’s basically what I expected after a couple of sessions and with one that focuses you on a picture and you look at the picture, and they go through the picture and everything, and it makes you more aware of your surroundings. It gives you more of a perspective of yourself too, of how to learn how to handle situations and you just use your thoughts, you use your senses, you use focusing (02 admin asst age 40-60, <5 sessions, applied, wave 1)  [preferred breathing over imagery]  I participated in two sessions. One session I found it very beneficiary when it was asked to get into a comfortable quiet position and taught some breathing techniques and concentration techniques. I found it very, very helpful. The other session I participated in, it was actually a picture. The teacher was just asking us to concentrate on that picture and I didn’t find it helpful at all so I actually left that. It didn’t give me any benefits. I didn’t like the concentration of the art. I didn’t find the connection. It didn’t give me good strategies. I didn’t like it (48 physiotherapist age 40-60, <5 sessions, not applied, wave 2)  [referring to breathing] One, was that I was able to find a quiet space in my own home and concentrating on the instructions, the position, the breathing techniques. Very soothing. The instructor was really soothing, really great. I didn’t want it to end [laugh] to be honest with you. I was able to follow all the recommendations and breathing. I really found it helped me a lot. It was closer how I was being taught before or what I also experienced before. Those kind of relaxation techniques were beneficial to me (48 physiotherapist age 40-60, <5 sessions, not applied, wave 2)  [breathing]  I found the breathing exercises helpful. Yes, I found the focus was helpful…Helping me to tune out everything outside and to just focus on my well-being, my body, my emotions for a little while (14 manager age 40-60, <5 sessions, not applied, wave 1)  [scientific mechanism of action]  <Name> will often give a theoretical explanation as to what is happening and what this approach does. And by having that extra information, it’s really helped me appreciate and make sense as to why this is an important practice (06 social worker age 40-60, 10+ sessions, applied, wave 1)  [self-care]  My personality is that I always helped other people, family. That’s me. That’s my job and that’s also my personality. I always put everybody in front of me instead of taking care of myself. Which I think I do have to change a little bit as I am getting older, I realize. Somehow that is really hard for me. This kind of session in a really, quotation mark, if I am forced to get into a place where I am the center and not everybody else but just me only, I think that would help me tremendously. I think I feel at this stage that I am recognizing it but feel I cannot do it on my own. I am still looking forward to help people instead of myself (48 physiotherapist age 40-60, <5 sessions, not applied, wave 2)  [Soft front/strong back, counting]  There is one, I think it’s soft front, strong back, so that one I’ve used. And the counting, you are breathing and you just count 1 in, 1 out, 2 in, 2 out, 3 out and then you just repeat (79 other age 40-60 applied wave 3) |
| Soothing voice of session facilitators |  | I find that Dr. <name> in particular has a really calming voice. I find that really helpful to get into a calm space (22 psychotherapist age <40, <5 sessions, applied, wave 2)  [about another facilitator] But having a very soft calming voice and a very relaxing sort of rhythm, cadence to her voice. She would pause after every two or three words but it felt natural. It wasn’t like you were left hanging like oh what is she saying after that. That was really nice (22 psychotherapist age <40, <5 sessions, applied, wave 2) |  | She just has a very soothing voice and I like her style and I like these mini practices that I have found incredibly helpful for work (79 other age 40-60 applied wave 3) |
| Reassurance and reminders offered by session facilitators |  | And she does a lot of reassuring throughout the sessions. Each time reminding everyone you do not need to turn on your video if you don’t want to, you don’t have to talk in the chat. Oh sorry, you don’t have to turn on your mic if you don’t want to, you can type in the chat. She also reminds us that there is no perfect way of doing what she is asking us to do and just try your best. And normalizing that it’s natural for your mind to wonder and stay curious of where it’s wandering to. That was really nice for someone who practicing mindfulness is new for (22 psychotherapist age <40, <5 sessions, applied, wave 2) | They were not judgmental. I would ask stupid questions in the beginning, they would always address them nicely, it felt welcoming  (35 physician age 40-60, #sessions, applied, wave 3) |  |
| Facilitated by experienced mental health professionals |  |  | I like the people, they are amazing psychiatrists, it felt comforting that there was a mental health professional doing it (35 physician age 40-60, #sessions, applied, wave 3)  Well I think they’re all very experienced and I found a bit of connection or appreciation for their approach. They all brought a calm and humble presence (75 physician age 40-60, # sessions, applied, wave 3) |  |
| Evening sessions | I think that the program was very responsive to feedback they got along the way because I noticed the times that they are offered had shifted slightly. They used to be 8 and 9 o’clock and then I guess now a lot are at 8.30… I also think it’s a great time of night… There is no way I could have made an 8 o’clock one every night… But the 8.30 change was really helpful for me (04 nurse practitioner age <40, 5-10 sessions, applied, wave 1)  For me the time has been pretty convenient. You are never going to have a time that works for everyone. I think in the beginning it was 9 o’clock and then, there was a phase where there was two, there was an 8 o’clock or 9 o’clock. That was kind of nice because you had the flexibility. And now it’s 8.30. But in general, for me those times are good because usually you’ve come home, you’ve already eaten your dinner so it’s kind of a nighttime routine. For me, it was usually pretty easy to fit in your schedule (01 physician age <40, 10+ sessions, applied, wave 1) | For me it was perfect. I wouldn’t change anything (10 physiotherapist age <40, 5-10 sessions, applied, wave 1)  I think it’s a really ideal time of day the 8.30pm time slot. It’s just late enough without feeling too late (22 psychotherapist age <40, <5 sessions, applied, wave 2)  As for the 8.30pm time slot, I personally would also entertain an even later slot like a 9 pm slot. Just because I really like the close to bedtime aspect of it but I could see why others might prefer 8.30pm instead. Especially if they’re facilitating it, they don’t want to go too late (22 psychotherapist age <40, <5 sessions, applied, wave 2)  The timing is great, actually. It’s an ideal time because I can make it. It’s far enough into the evening that I can get things done before it begins and it’s late enough that I could possibly use it as a transition to sleep. That’s the perfect time. I hope it continues at the same time (46 psychotherapist age <40, <5 sessions, applied, wave 2) | It’s hard for us to get to it any earlier. I think the timing is perfect (44 physician age 40-60, <5 sessions, not applied, wave 2)  It’s really the perfect time because it’s right after dinner is done, I can move around for a couple of minutes, do this and then often get back to doing some work after between 9 and 10. So for me the timing is perfect (75 physician age 40-60, # sessions, applied, wave 3)  I’m usually in bed at 9.30 to get up at 5.30 so the timing for us was perfect (78 physician age 40-60 #sessions applied wave 3) | The time is perfect because that is the time you get home from work, you have your dinner and it is more in the relaxed mode, you do the session and then it’s like ok now it’s time for me to just chill, relax, not do anything else and then just focus and you know relax before going to bed (02 admin asst age 40-60, <5 sessions, applied, wave 1)  It’s a good time of the day… I’m glad mindfulness is in the evening and I think it’s just a good fit with my routine (06 social worker age 40-60, 10+ sessions, applied, wave 1)  Doing it after hours is nice. If it happened during the day, I would sign up and then there is something else that is more important and I’ll do work instead of that. Having it in the evenings, I can take a break from emails and focus on this (14 manager age 40-60, <5 sessions, not applied, wave 1)  I loved the session to be taught in the evening and I can go you know two times a week…For me, the 8.30 evening session was working because I am home and I can get away from my family and find a quiet place. If it was being offered daytime, I would not be able to participate because at my workplace I will not be able to do it (48 physiotherapist age 40-60, <5 sessions, not applied, wave 2) |
| 30-minute session | Having anything any longer would be maybe too much of a commitment and then anything shorter I don’t know if you could like get into it (04 nurse practitioner age <40, 5-10 sessions, applied, wave 1)  I think 30 minutes is kind of a sweet spot…For me, I think it is actually a nice amount of time. If it is an hour, I feel that is a harder commitment to make, and then if it’s 15 minutes, that’s just really short. I think that it’s actually the perfect amount of time (01 physician age <40, 10+ sessions, applied, wave 1) | 30 minutes is really good. It’s long enough to settle and really engage with the process or with the practice. But not so long that it feels like a greater time commitment that is harder to fit in. I think 30 minutes was perfect (10 physiotherapist age <40, 5-10 sessions, applied, wave 1)  I don’t think I would want it to be much longer, especially for an introductory setting for a lot of folks who may not have any experience with getting into a mindful meditative state. I think that 20-to-30-minute mark is inviting and unintimidating (22 psychotherapist age <40, <5 sessions, applied, wave 2)  Yes, it’s perfect (46 psychotherapist age <40, <5 sessions, applied, wave 2)  I think 30 minutes is a pretty decent timeline because it’s good for beginners, if you’re just learning about mindfulness, 30 minutes is a long time, right? So I think 30 minutes is good to get people involved and being proud of themselves and feeling accomplished with what they did, and then if people have more mindfulness experience they can just continue on their own outside of that (76 admin assist, age <40, # sessions, applied, wave 3) | Yes, I think it sounds good (44 physician age 40-60, <5 sessions, not applied, wave 2)  I like that it’s a half an hour into the evening…it’s not a big time commitment…they found the perfect length (35 physician age 40-60, #sessions, applied, wave 3)  I find that it’s long enough to have an impact, but I think if it was longer, it might actually be harder for me to commit to attend (75 physician age 40-60, # sessions, applied, wave 3)  The 30 minutes disappeared, they tended to go by very, very quickly. I think for the goal of the program 30 minutes was good. I’m not sure less time would have got me where I needed to be…and the facilitators were super at running it and staying on time and keeping everything moving forward (78 physician age 40-60 #sessions applied wave 3) | They do half an hour. It is perfect because that time you can always fit in. If sometimes it is a little bit longer than you just can’t really focus as much (02 admin asst age 40-60, <5 sessions, applied, wave 1)  It’s not a large time commitment, it’s half an hour. Thirty minutes is certainly enough to be able to get the benefits for me of the mindfulness exercise (06 social worker age 40-60, 10+ sessions, applied, wave 1)  I have done 10-minute sessions and that was nice. I could fit it in but I think actually taking a half hour, that feels really substantial. Like I’m doing something (14 manager age 40-60, <5 sessions, not applied, wave 1)  It did end a little bit earlier like 25 minutes which I probably would prefer 30 minutes like total relaxation. I understand maybe longer will be way too much (48 physiotherapist age 40-60, <5 sessions, not applied, wave 2)  The 30 minutes was perfect (79 other age 40-60 applied wave 3) |

**Barriers**

| Theme | Age <40 | | Age 40-60 | |
| --- | --- | --- | --- | --- |
|  | Clinicians (physicians, nurse practitioners) | Allied health and other (physiotherapist, social worker, psychotherapist, admin asst, manager) | Clinicians (physicians, nurse practitioners) | Allied health and other (physiotherapist, social worker, psychotherapist, admin asst, manager) |
| **To participating in sessions** | | | | |
| Making it a priority, finding time | Main challenges was probably just my own stuff, staying focused, making it a priority, come. I’ve only ever gone on Monday and Tuesday evenings and I would like to just make a priority to go on a Wednesday and Thursday and just get exposed to some different leaders (04 nurse practitioner age <40, 5-10 sessions, applied, wave 1)  Making it a priority and committing to it. I think it’s probably [inaudible] your devotion to something like this. That’s probably been a challenge making it a priority and making it part of a routine which is something I council people on doing all the time and then we don’t do it personally (04 nurse practitioner age <40, 5-10 sessions, applied, wave 1)  Trying to stay consistent and participate as much as I could. Sometimes I actually wanted to try more of the other days, I usually do the Thursdays, and I was trying to do the other days but that didn’t always really happen. When you are busy, scheduling itself can be a challenge (01 physician age <40, 10+ sessions, applied, wave 1)  I guess the time of day. Although it is very convenient for me to attend, I do have to make a choice to do. Either I fit in time with my husband, or I prep for tomorrow or clean up. It’s a choice. You have to make a choice to go cause there’s always something else that you could be doing (04 nurse practitioner age <40, 5-10 sessions, applied, wave 1) | Making sure to set aside the time and prioritize this type of practice or self-care activity amidst some competing priorities for time and energy… the time and energy being devoted to just doing this practice for 30 minutes and not doing something else (10 physiotherapist age <40, 5-10 sessions, applied, wave 1)  I’m distracted, I don’t prioritize it, I forget how helpful it is, I forget. For whatever reason I don’t prioritize it (46 psychotherapist age <40, <5 sessions, applied, wave 2)  It’s hard to get to because life is busy and so I try to attend and make the time but sometimes I am not able to (76 admin assist, age <40, # sessions, applied, wave 3)  I think the sessions are at a good time like Tuesday and Thursday night in the evening but sometimes I do have plans that come up and other things that I want to do like go out with my friends or whatever (76 admin assist, age <40, # sessions, applied, wave 3) | I guess it’s just the time. You’re not making the time to do it. I think that’s really what it comes down to (44 physician age 40-60, <5 sessions, not applied, wave 2) | And just trying to find time in my day to do it (14 manager age 40-60, <5 sessions, not applied, wave 1)  Sometimes it’s just committing the time to it. Scheduling your day to be able to do it (06 social worker age 40-60, 10+ sessions, applied, wave 1)  It’s tricky, that time of night, so I actually set my alarm on my phone to go off to remind me to go, because I get up really early for work so that time doesn’t always work the best for me (79 other age 40-60 applied wave 3) |
| Being tired, easily distracted | Some days you are more into it and some days you’re very distracted and tired and kind of sitting there and feeling like you are not getting quite as much out of it, but I wouldn’t say there was ever a time where it doesn’t help at all (01 physician age <40, 10+ sessions, applied, wave 1)  Sometimes you are very distracted and it’s hard to focus, it’s hard to sit still, it’s hard to stay with the practice. And then especially when we have our cameras off, sometimes I could just get up or I could check my phone, no one knows. Whereas in the beginning when we had our cameras on you really feel like okay I have to really sit still because if I’m moving around people are going to see. But like I said, that’s not really a challenge of the program I think it’s more specific to any form of meditation practice (01 physician age <40, 10+ sessions, applied, wave 1) | As always a busy mind and very active brain makes it a challenge but I realize is part of the process (10 physiotherapist age <40, 5-10 sessions, applied, wave 1) |  | The main challenges was trying to remain focused and not letting your mind wander elsewhere. At the beginning that was the hardest challenge for me because you start thinking about what am I going to do tomorrow for work and what’s my schedule like for tomorrow. That was the most challenging part, trying to get your mind not to think ahead and try to focus on the session and try focusing on the here and now which I am better at doing now than when I first started (02 admin asst age 40-60, <5 sessions, applied, wave 1) |
| Prefer some practices over others | There is just certain times where it is going to speak to you a little bit more whether it’s a particular theme or exercise, sometimes it resonates more and sometimes it doesn’t (01 physician age <40, 10+ sessions, applied, wave 1) | [keeping eyes open for entirety of imagery exercise reduced relaxation]  The psychiatrist that ran the session last night was suggesting to keep our eyes open and engage with the painting. And that was a little bit distracting for me especially the unnatural blue light coming off the screen. I was in a dark room trying to get to a quiet peaceful place so instead I just looked at the picture for a minute and tried to take everything in and close my eyes and visualize the painting. And followed her instructions of looking at the figures and the colors and the waves through my mind’s eye. That was much more relaxing for me than keeping my eyes open looking at the harsh light of the screen in a purposeful dark room that I had made. But nonetheless I enjoyed the painting exercise (22 psychotherapist age <40, <5 sessions, applied, wave 2)  They were doing a mindful observation of a painting, and it looks like a really classical painting, some European fancy artist or something like that, and I actually didn’t really like that practice very much. I strongly identify as being First Nation and Ojibwe. In mainstream Western culture, things that are held up as being the norm, but for me, it’s not actually soothing, it reminds me of colonization and trauma (76 admin assist, age <40, # sessions, applied, wave 3) | I suspect one method doesn’t work for everyone…it’s hard to do and to learn and process… to use the visual and to work through the pictures, I don’t’ know, it didn’t give me the same feeling, I couldn’t get to the same place (78 physician age 40-60 #sessions applied wave 3) | [poetry]  In the beginning, I did not like the use of poems or self-compassion exercises. I reflected that I hated poetry from high school. But I’ve come to appreciate that. The very short aspects of poetry that are used are really reflective of the mindfulness exercises that we are doing. I really started to enjoy those reflections and I’ve come around (06 social worker age 40-60, 10+ sessions, applied, wave 1)  Most of the sessions are where you would do a concentrative piece, and then you break, and then sometimes there’s a question and answer, and then you go back and do another little mini practice. That piece I found very disruptive because once you are kind of in that zone of being a bit calmer and you are thinking about whatever it is that they are talking about, to have it break off in the middle and then go back and do another little session, I didn’t like that. I’d rather they move right into a mini session and then do q and at the end (79 other age 40-60 applied wave 3)  She references paintings pretty much every time and so you’re thinking about this portrait or this painting, and it just doesn’t resonate with me, but obviously everyone is different (79 other age 40-60 applied wave 3) |
| Virtual format may lessen full benefit |  | There are some barriers to doing it virtually and not sharing the same physical space with somebody. I think it is a limitation (46 psychotherapist age <40, <5 sessions, applied, wave 2) |  |  |
| Concerned about being seen by others in vulnerable state |  | The one thing did cross my mind, this is a time I was hoping to be in my pajamas, wash my face, maybe lying down, and I wondered am I going to have to be on camera? I don’t want other people to see me in this kind of vulnerable state but that was quickly cleared up at the beginning of the Zoom call (22 psychotherapist age <40, <5 sessions, applied, wave 2) |  | It’s weird because it’s online…the hesitation would be showing my video images on the screen, even though I would show up to a live group session with other colleagues, that didn’t bother me, but for some reason being online bothered me…I just prefer being anonymous online (79 other age 40-60 applied wave 3) |
| Unstable Internet connection | Sometimes the audio or even the video, it gets a little frozen. It didn’t happen a lot but there was a couple of instances where the sound would get kind of echo-y or where the image on the screen would freeze for a couple of seconds. So more of a technical challenge but it was never to the point where it was that disruptive (01 physician age <40, 10+ sessions, applied, wave 1) | It would be internet connection, which is rare, but that would be a challenge. There would be no other way for me to sign on because cellular service is poor. We’re in a pretty rural area so that would mean I am not able to access the practice, even if I set aside a time specifically for it. That could be a barrier. It hasn’t been but that could be (46 psychotherapist age <40, <5 sessions, applied, wave 2) | One night we couldn’t get signed on, but that problem was on our end (78 physician age 40-60 #sessions applied wave 3) |  |
| Finding quiet space at home |  | Finding a quiet space in the house. I have to be really intentional about that part of things. I really need to set that up. Versus going and attending a class. Well, it involves transportation, parking and all of that. It is very much contained to a room, to a classroom, so than it’s easy once you get into that space. But now, even if I get in the room, even if I close the door and set it up, there’s still this possibility of other sounds in the house or somebody coming in. I think that’s a barrier to full participation, a distraction (46 psychotherapist age <40, <5 sessions, applied, wave 2)  I’m picturing a yoga class or meditation class held at a studio… I plan the week around that… There’s more intention and more energy, more effort in getting to a physical space. I think there’s value in that. I didn’t notice that until I didn’t have any of it…I’m comparing it to sitting in the same chair that I am working in, in the same room that I do my work in, and that being unchanged [from] the room where I would participate in a session (46 psychotherapist age <40, <5 sessions, applied, wave 2) |  |  |
| Not restricted to healthcare workers | If people did want to speak up or share something about their clinical experiences these days on the frontlines, people might feel more comfortable if they know the other people are definitely providers, not just some random strangers. Logistically, I understand that would be cumbersome, you need people to create a login, and that brings up its own issues. In the beginning, I felt more confident that it was actually healthcare providers. So I felt that was more secure. But now there’s a website that’s open to anyone, anywhere in the world, and with everyone having their cameras off and themselves on mute, you don’t know who people are. I wonder if that actually might be a bit of a barrier to people feeling comfortable to ask a question or chime in with some insights or feedback (01 physician age <40, 10+ sessions, applied, wave 1)  Early on I think that their numbers were predominantly healthcare providers, like front line healthcare providers. Then as it evolved, I feel they opened it up to sort of anyone working on the frontline and in any capacity and so I felt that the targeted intervention, or messaging to healthcare providers, kind of changed. Which wasn’t necessarily bad, but I just noticed that over time they were inviting a larger audience (04 nurse practitioner age <40, 5-10 sessions, applied, wave 1)  If one of the goals of the intervention was more engagement or discussion, or people sharing their stories, keeping it to the regulated professionals might have been more of a safe space, I guess. Not knowing who was on the call. I think at times kind of kept me from speaking up or asking questions or participating in discussions or feedback. Whereas, if it was just my peers, then it may have felt more of a safer space to talk (04 nurse practitioner age <40, 5-10 sessions, applied, wave 1) |  |  |  |
| **To adopting or using mindfulness** | | | | |
| Forgetting or unable to invoke the skill |  | Forgetting and maybe not having such an automatic, not being so practiced that it’s automatic with using that skill (10 physiotherapist age <40, 5-10 sessions, applied, wave 1)  Just remembering. Remembering to do so. It hasn’t been something that I have been trained to do so it takes a lot more effort and add-on to implement these new learned behaviors (22 psychotherapist age <40, <5 sessions, applied, wave 2) | Finding the time (78 physician age 40-60 #sessions applied wave 3) | Also, when feeling really anxious and hyper involved with something, just to recognize that there is a way to step back, and not be as conflictive and distressed about something. Having that awareness that there are options (06 social worker age 40-60, 10+ sessions, applied, wave 1) |
| Finding a quiet space at work |  |  |  | My problem is that I cannot find a quiet space to do it during the daytime. I can practice the breathing and the relaxation techniques but to find the position. I was lying down which helped me a lot and to find a quiet space, that’s the challenging part (48 physiotherapist age 40-60, <5 sessions, not applied, wave 2) |

**Enablers**

| Theme | Age <40 | | Age 40-60 | |
| --- | --- | --- | --- | --- |
|  | Clinicians (physicians, nurse practitioners) | Allied health and other (physiotherapist, social worker, psychotherapist, admin asst, manager) | Clinicians (physicians, nurse practitioners) | Allied health and other (physiotherapist, social worker, psychotherapist, admin asst, manager) |
| **To participating in sessions** | | | | |
| Facilitated rather than self-directed | If I miss it she is going to know I wasn’t there so it’s like having a personal trainer. Whereas if I’m meditating by myself if I don’t do it nobody actually knows (01 physician age <40, 10+ sessions, applied, wave 1) |  | What really helped was the fact that we were being led through the whole thing. What helped was the entire session Dr <name> was guiding you through. That’s the only way I could do it right now. If I was doing it just myself for half an hour, I don’t think I could do it (44 physician age 40-60, <5 sessions, not applied, wave 2)  There was always someone there that could guide me through the mindfulness (35 physician age 40-60, #sessions, applied, wave 3) | Right now, I think I will need somebody with me to instruct me how to do it like a coach, like a teacher. I think I would like to have somebody to be with me and tell me (48 physiotherapist age 40-60, <5 sessions, not applied, wave 2)  if it’s not scheduled for me then it just won’t happen. Okay, it happens at a certain time, I’m going to log on and join the group for that time. It’s more like a habit that way (14 manager age 40-60, <5 sessions, not applied, wave 1) |
| Virtual, easy to participate |  | I like that it’s online because I think it makes it more accessible both time-wise, schedule-wise and all of that, and it helped to ensure the anonymity piece. As well, I have struggled with anxiety, so sometimes showing up in person to things like that, it can actually be a barrier that triggers anxiety for people, so I actually liked the format of it (76 admin assist, age <40, # sessions, applied, wave 3) | It’s convenient because it’s online, you don’t have to go somewhere, you can just Zoom in. It’s hard to get myself going somewhere to an actual place, but it is good to be with people. You kind of feel like you are with them, even if you don’t see them, so for me, the virtual format was perfect (35 physician age 40-60, #sessions, applied, wave 3)  It was always at a fixed, predictable time, so you can plan your schedule around it (78 physician age 40-60 #sessions applied wave 3) | It’s actually quite nice because it’s so convenient, right? You just jump online and join. It’s really convenient because you can do it from anywhere. And they are on the screen, so if you’re not sure what they’re talking about, you can look at your screen and see what they are doing (79 other age 40-60 applied wave 3) |
| Well-designed web site with link to sessions | In the beginning, it was just Zoom link, but Zoom links are really hard to remember because they have all these letters and numbers, and so I’d have to go through my email to find that bulletin that had the link. Now you can just go to the website and click directly so that’s definitely made it easier (01 physician age <40, 10+ sessions, applied, wave 1) | The website was really clean and actually that’s one the reasons I probably pursued it in the end. I thought it looked professional, really clean, uncluttered, organized. I think that’s what actually got me to do it in the end because there are a lot of other services that either describe something similar or that you can sign up for (46 psychotherapist age <40, <5 sessions, applied, wave 2) |  |  |
| Anonymity | I did think a little bit about the anonymity and confidentiality of it whether we would be encouraged to turn our videos on or identify ourselves. It doesn’t necessarily bother me, but I just thought it would have maybe been a barrier for others (04 nurse practitioner age <40, 5-10 sessions, applied, wave 1) | I think that’s a really powerful aspect of the program that invites more people to attend because you can change your screen name and be anonymous so I thought that was cool. I think that anonymity aspect is really important so that everybody can just feel safe to access support and not have to reveal their identity from fear of judgement from coworkers (76 admin assist, age <40, # sessions, applied, wave 3) | It’s anonymous, so you don’t have to put your video on and you don’t have to talk if you don’t want to, you just drop in (35 physician age 40-60, #sessions, applied, wave 3) |  |
| Shared activity with other healthcare workers |  | I think it just helps to have a shared practice or shared experience with someone that I work with in my field and respect. I think that’s a benefit. It’s a small community support outlet (10 physiotherapist age <40, 5-10 sessions, applied, wave 1)  This one seemed like the best suited, professional and the healthcare specific work (46 psychotherapist age <40, <5 sessions, applied, wave 2) |  |  |
| **To adopting/using mindfulness** | | | | |
| Techniques easy to adopt |  |  |  | They’re just more readily available. In my office, I have a picture on the wall. If I get really stressed at work, I can sit there and I can take a look at that picture and study it. It’s just something a little bit more accessible for me. Same thing, the senses, that’s readily available, just sitting there and breathing (02 admin asst age 40-60, <5 sessions, applied, wave 1) |
| Advice and examples on how to integrate practice in daily life |  | The way the facilitators are able to link the practices to real, realistic, day-to-day moments where the skills might come in handy. I think they show a real, genuine humanity when they talk about how these practices might apply. Because they may give personal examples, validate or share many ways in which many people might feel stressed in a given moment. There is that kind of normalizing stresses and providing some very practical mindfulness-based skills for coping with them. It’s not very concise but yes, I think the suggestions, gentle reminders of where these skills can be useful in daily life (10 physiotherapist age <40, 5-10 sessions, applied, wave 1) | I like the tips around suggestions for what to do, practices that you can take away and try at a different times. Hearing about the different possible ways of using mindfulness in the moment during the day is helpful (75 physician age 40-60, # sessions, applied, wave 3) |  |
| Web site enables practice outside of facilitated sessions |  |  | The website is good, where they have a couple of little movement things (35 physician age 40-60, #sessions, applied, wave 3) | The website… certainly does give a few more options in terms of if I wanted to do more of a formal practice on the weekend or on a time of the day when the live stream isn’t available. The four groups and the website being available with the other practices it’s a good combination (06 social worker age 40-60, 10+ sessions, applied, wave 1) |

**Recommendations**

| Theme | Age <40 | | Age 40-60 | |
| --- | --- | --- | --- | --- |
|  | Clinicians (physicians, nurse practitioners) | Allied health and other (physiotherapist, social worker, psychotherapist, admin asst, manager) | Clinicians (physicians, nurse practitioners) | Allied health and other (physiotherapist, social worker, psychotherapist, admin asst, manager) |
| **To increase or support participation** | | | | |
| Wider advertising to bring benefit to more people |  | Just spreading the message. When I log on, I feel a little bit deflated when I see 6 people, 8 participants, 9 participants and I think oh my gosh I know there are so many more people that would benefit from this throughout the country. How is there only a couple handful of people participating? I certainly try to spread by word of mouth. But I don’t know if there could be a flyer blasted out to every family health team, family health organization in the province for folks to know to sign on. Because it feels deflated, but then I almost feel greedy and selfish. Why do I get to partake in this and others aren’t? I know there are others that would enjoy this and need it and would benefit from it (22 psychotherapist age <40, <5 sessions, applied, wave 2) |  |  |
| Inform in advance that camera and microphone will be off (to alleviate concern) |  | The sort of, I’ll call it unnecessary, anxiety of am I going to have to be on camera during this. I think it would of been helpful on the website to just put a little disclaimer. Maybe on the ‘view live schedule’ right where you click ‘join’ like an asterisk, your camera and mic are automatically turned off for the session. Because I noticed they are. And then you can turn them on if you wish. So that than I wouldn’t have to have that oh gosh am I going to be on camera, is someone going to be watching me, do I have to still be in my work clothes or can I just be in my pajamas ready for bed (22 psychotherapist age <40, <5 sessions, applied, wave 2) |  |  |
| Reminder on day of session |  | It’d be neat to send some kind of reminder, maybe a reminder email, you know tonight is the Pause 4 Providers at 8.30. Or text. While I am invaded with emails and messages all day long, I think it would actually be useful because it would touch on my to-do list one more time. It would just highlight this is coming up. Even if I would just delete the email. I think one reminder would even increase the likelihood even more that I would attend (46 psychotherapist age <40, <5 sessions, applied, wave 2) |  |  |
| Advance notice of techniques that will be practiced during forthcoming session |  |  |  | More of the session that I had more of the relaxation, breathing, concentration techniques instead of the art. I don’t know is that always like that? One is a relaxation and one is the art? (48 physiotherapist age 40-60, <5 sessions, not applied, wave 2) |
| More sessions at different times to suit various schedules |  | Maybe one more on the weekend, that would be good. Could be every second or every third week. I think that would be good. That would be helpful. I would attend (46 psychotherapist age <40, <5 sessions, applied, wave 2) | If you’re trying to extend your reach, have a menu to choose from, so if people have a couple of different time slots they could sign up for, it’d be easier to plan around (78 physician age 40-60 #sessions applied wave 3) | It would be helpful to have more sessions available depending on people’s schedules (14 manager age 40-60, <5 sessions, not applied, wave 1)  Maybe the more frequency instead of twice a week, keeping it half an hour. Maybe that would be something to look into (48 physiotherapist age 40-60, <5 sessions, not applied, wave 2)  Maybe the other option could be weekend as well (48 physiotherapist age 40-60, <5 sessions, not applied, wave 2)  I would love if it was available every night. It’s a nice calming ritual before people go to bed instead of looking at their phones or watching the news (14 manager age 40-60, <5 sessions, not applied, wave 1) |
| Larger cadre of facilitators to increase variety |  | There could be a variety of instructors maybe. That’s the only thing I can think of. Maybe if there was another class if there was a third class there could be another instructor just to keep the variety (46 psychotherapist age <40, <5 sessions, applied, wave 2) |  |  |
| Restrict to healthcare workers, or enable teams or similar professions to practice together | I would probably buy in a little bit more, if I knew that it was just healthcare providers. I would find that just a little more of a community. Just knowing that everyone was you know either a doctor or a nurse. just to know that people were doing the same kind of work, encountering the same kind of organizational things and healthcare things than I do (04 nurse practitioner age <40, 5-10 sessions, applied, wave 1) |  |  | Maybe a breakout room would be helpful. I would love to be able to do it with my team somehow. For all to be able to take half an hour and say ok we’re going to do this together. We’re a small team, there’s like 16 of us. I just think it would be so nice to just have our team just take half an hour and focus on their wellness… it would be nice for us to be able to talk together if there was a breakout room (14 manager age 40-60, <5 sessions, not applied, wave 1)  I work for <department> and I know that there are <similar departments> at some of the other hospitals as well so it might be to compare notes with other people who do a similar job to me. I can’t really relate to somebody who is in the ICU caring for COVID patients so I wouldn’t want to my equate my experience with theirs (14 manager age 40-60, <5 sessions, not applied, wave 1) |
| Specify agenda at outset of each session (to prime for length of exercises) | Perhaps setting up a what to expect 30 seconds at the beginning, like this is going to be the format of the session. Because I found that sometimes in the longer practices I would actually, and they are constantly reminding you that your mind will wander and that is part of mindfulness to imagine that you’ve wondered and bringing it back]. Sometimes when my mind would wander to how long is this actually going to be, is this a 3-minute thing, is this a 12-minute thing, should I readjust my posture sit more comfortably, for how long am I in this for. Just more of an agenda would be helpful (04 nurse practitioner age <40, 5-10 sessions, applied, wave 1) |  |  |  |
| Spend more time on fewer practices per session |  |  |  | I actually would prefer that we not extend the time, 30 minutes is good, but do longer pieces because they never felt that long like maybe 10 or 15 minutes or something that you would be working on one thing. So I found that it wasn’t long enough for me (79 other age 40-60 applied wave 3) |
| Encourage acceptance of emotions |  | Just encouraging everybody to be really accepting whatever the emotions are that they are having, like it’s normal to cry or whatever, right? Because we’ve all had different experiences, so I would say probably incorporating trauma-informed principles a little bit more would be awesome to just really encourage people to feel their feelings (76 admin assist, age <40, # sessions, applied, wave 3) |  |  |
| Greater interactivity | I was hoping that there would be something, not interactive, you don’t want to sit there and talk to a bunch of people, and that would certainly add on to the time. I would say when everyone’s camera is off and everyone is mute, then when there is an invitation for feedback or question, no one says anything, and no one says anything at the end. Sometimes it would be nice if people could chime in a bit more but I don’t know how. I think people are shy, myself included, I’m not quite sure how that hump can be overcome. Sometimes, if one person says something, then a few other people will chime in, but it’s tough because every week it’s a drop in, so there aren’t the same people, so there isn’t that level of familiarity. I don’t know if people could be encouraged to put their camera on even for a few seconds in the beginning. I understand not wanting to have your camera on while you’re actually sitting there meditating because then you’re just sitting in front of the screen with your eyes closed and you might feel self-conscious that people are looking at you. But maybe in the beginning, just to welcome people and see who else is there, see a friendly face, that type of thing. It’s kind of nice. Sometimes I would like it to be a little bit less anonymous. But I guess some people they might like it because it’s really anonymous. Sometimes you just wonder who else is there and where other people are coming from, what’s their specialty, what’s their situation. Not in a nosy way but just you wonder who you are sitting with virtually (01 physician age <40, 10+ sessions, applied, wave 1) | It would be cool to hear more about their work. You know, I’m so and so, this is my background and this is what I have been doing lately (46 psychotherapist age <40, <5 sessions, applied, wave 2) |  |  |
| **To enable adoption/use of mindfulness** | | | | |
| Quiet space at work to practice mindfulness |  |  |  | If I can do the session here at the workplace, provide me with a quiet place where I can just be on my own finding a position like lying down or sitting. I prefer lying down where my body can totally relax and my mind can focus on myself. It’s the lack of area where I can practice this during the daytime (48 physiotherapist age 40-60, <5 sessions, not applied, wave 2) |
| Provide self-directed reinforcing material or refer to other resources | Perhaps more self-directed resources or if the sessions were recorded and you could listen to the same one again, that would be kind of nice… there is certainly certain sessions where I was like oh that was a really good one and if I was able to, I probably would of listened to it again at a different time (04 nurse practitioner age <40, 5-10 sessions, applied, wave 1) | I think they have some resources that they might put up on an Instagram page or something. And maybe they put it on the website too. I don’t know. Given that I don’t check Instagram very often, maybe if there are some cues or resources that would help someone use, be able refer to and self-guide a similar practice. I don’t know if that is something that makes sense to do but it may be something to think about. Some easy reference like practice support guide or something. I think they do post some things and maybe they are already there (10 physiotherapist age <40, 5-10 sessions, applied, wave 1) | Maybe having a list of some kind of different mindfulness exercises on the website may be good. It would be good to have some kind of resource, like, all the different approaches, a list of different meditations, something look at to know, okay, I understand this one now (35 physician age 40-60, #sessions, applied, wave 3) | I understand that it was a 6-months pilot. I appreciate that it can’t continue on forever, but I am thankful for the availability. I would assume at some point, and maybe even on the website, there are other resources or other podcasts, that they recommend for people to continue on with the practice, if the program needs to discontinue (06 social worker age 40-60, 10+ sessions, applied, wave 1) |
